# Plyometric-Jump Training Effects on Physical Fitness and Sport-Specific Performance According to Maturity: A Systematic Review with Meta-analysis

**DOI:** 10.1186/s40798-023-00568-6

**Published:** 2023-04-10

**Authors:** Rodrigo Ramirez-Campillo, Andrew Sortwell, Jason Moran, José Afonso, Filipe Manuel Clemente, Rhodri S. Lloyd, Jon L. Oliver, Jason Pedley, Urs Granacher

**Affiliations:** 1 Exercise and Rehabilitation Sciences Institute, School of Physical Therapy, Faculty of Rehabilitation Sciences, Universidad Andres Bello, 7591538 Santiago, Chile; 2 School of Nursing, Midwifery, Health Sciences and Physiotherapy, University of Notre Dame Australia, Sydney, Australia; 3 School of Sport, Rehabilitation and Exercise Sciences, University of Essex, Colchester, Essex CO43SQ UK; 4 Centre of Research, Education, Innovation, and Intervention in Sport, Faculty of Sport, University of Porto, Porto, Portugal; 5 Escola Superior Desporto e Lazer, Instituto Politécnico de Viana do Castelo, Rua Escola Industrial e Comercial de Nun’Álvares, 4900-347 Viana do Castelo, Portugal; 6 Research Center in Sports Performance, Recreation, Innovation and Technology (SPRINT), 4960-320 Melgaço, Portugal; 7 Instituto de Telecomunicações, Delegação da Covilhã, 1049-001 Lisbon, Portugal; 8 Youth Physical Development Centre, Cardiff School of Sport and Health Sciences, Cardiff Metropolitan University, Cardiff, CF23 6XD UK; 9 Department of Sport and Sport Science, Exercise and Human Movement Science, University of Freiburg, Sandfangweg 4, 79102 Freiburg, Germany

**Keywords:** Plyometric exercise, Musculoskeletal and neural physiological phenomena, Human physical conditioning, Movement, Muscle strength, Resistance training, Youth sports

## Abstract

**Background:**

Among youth, plyometric-jump training (PJT) may provide a safe, accessible, and time-efficient training method. Less is known on PJT effectiveness according to the maturity status.

**Objective:**

This systematic review with meta-analysis set out to analyse the body of peer-reviewed articles assessing the effects of PJT on measures of physical fitness [i.e., maximal dynamic strength; change of direction (COD) speed; linear sprint speed; horizontal and vertical jump performance; reactive strength index] and sport-specific performance (i.e., soccer ball kicking and dribbling velocity) according to the participants’ maturity status.

**Methods:**

Systematic searches were conducted in three electronic databases using the following inclusion criteria: (i) Population: healthy participants aged < 18 years; (ii) Intervention: PJT program including unilateral and/or bilateral jumps; (iii) Comparator: groups of different maturity status with control groups; (iv) Outcomes: at least one measure of physical fitness and/or sport-specific performance before and after PJT; (v) experimental design with an active or passive control group, and two or more maturity groups exposed to the same PJT. The DerSimonian and Laird random-effects models were used to compute the meta-analysis. The methodological quality of the studies was assessed using the PEDro checklist. GRADE was applied to assess certainty of evidence.

**Results:**

From 11,028 initially identified studies across three electronic databases, 11 studies were finally eligible to be meta-analysed (*n* total = 744; seven studies recruited males; four studies recruited females). Three studies were rated as high quality (6 points), and eight studies were of moderate quality (5 points). Seven studies reported the maturity status using age at peak height velocity (PHV; pre-PHV values up to − 2.3; post-PHV up to 2.5). Another four studies used Tanner staging (from Tanner I to V). The training programmes ranged from 4 to 36 weeks, using 1–3 weekly training sessions. When compared to controls, pre-PHV and post-PHV participants obtained small-to-moderate improvements (ES = 0.35 − 0.80, all *p* < 0.05) in most outcomes (i.e., sport-specific performance; maximal dynamic strength; linear sprint; horizontal jump; reactive strength index) after PJT. The contrast of pre-PHV with post-PHV youth revealed that PJT was similarly effective in both maturity groups, in most outcome measures except for COD speed (in favour of pre-PHV). PJT induces similar physical fitness and sport-specific performance benefits in males and females, with a minimal exercise dosage of 4 weeks (8 intervention sessions), and 92 weekly jumps. Results of this meta-analysis are based on low study heterogeneity, and low to very low certainty of evidence (GRADE analysis) for all outcomes.

**Conclusion:**

Compared to control participants, PJT resulted in improved maximal dynamic strength, linear sprint speed, horizontal jump performance, reactive strength index, and sport-specific performance (i.e., soccer ball kicking and dribbling velocity). These effects seem to occur independently of the maturity status, as both pre-PHV and post-PHV participants achieved similar improvements after PJT interventions for most outcomes. However, several methodological issues (e.g., low sample sizes and the pooling of maturity categories) preclude the attainment of more robust recommendations at the current time. To address this issue, consistency in maturity status reporting strategies must be improved in future studies with the general youth population and youth athletes.

**Supplementary Information:**

The online version contains supplementary material available at 10.1186/s40798-023-00568-6.

## Key Points

- Plyometric jump training is an effective intervention to improve physical fitness of youth participants, including maximal dynamic strength, linear sprint speed, horizontal jump performance, reactive strength index, and sport-specific performance (e.g., soccer ball kicking velocity).
- Plyometric jump training induces similar physical fitness and sport-specific performance benefits in males and females, with a minimal exercise dosage of 4 weeks (8 intervention sessions), and 92 weekly jumps.
- Plyometric jump training is similarly effective in pre- and post-PHV youth in most outcomes, except for COD speed (in favour of pre-PHV).
- Results of this meta-analysis are based on a total of 744 participants, from 11 articles with moderate to high methodological quality, low study heterogeneity, and low to very low certainty of evidence (GRADE analysis) for all outcomes.

## Introduction

Physical fitness is correlated to current and future health outcomes [1–3]. There is additional evidence from original research that improvements in muscle power result in increased bone mass in school-aged participants [4]. Moreover, high levels of physical fitness appear to facilitate motor skill learning [5–8] in youths and adults and help to reduce the risk of sustaining musculoskeletal injuries [9–12]. Schools or sport clubs can provide excellent opportunities to deliver exercise interventions to improve physical fitness and sport-specific performance (SSP) because youth can be reached irrespective of their socio-economic background. Intervention programs implemented in schools or sport clubs should be easy to administer and afford little extra equipment [13, 14]. Accordingly, safe, effective and joyful training programs are needed that require little equipment and occupy large groups of individuals [15, 16], such as plyometric jump training (PJT).

Intervention programs involving PJT may provide an exercise mode that can be adapted for populations according to their age, sex, health, physical fitness and/or SSP level [17–20]. Indeed, PJT emphasises the use of jumping drills, such as hops, depth jumps, bouncing, or skipping exercises. Moreover, using limited or no equipment, it is possible to promote a wide range of exercises and intensities through the modification of variables such as drop height, ground contact time, direction (e.g., vertical vs. horizontal), type of surface (e.g., stiff- or soft-surfaces; horizontal vs. incline), landing type (e.g., bilateral vs. unilateral), or loading parameter (e.g., external load vs. unloaded; assisted vs. resisted). The implementation of PJT in schools or sports clubs may enhance physical fitness and additionally improve students’ perception of physical activity [21, 22]. Interventions using PJT have proven to be effective to promote markers of health such as bone mineral content [17, 23], measures of physical fitness (e.g., linear sprint speed) [8, 24–28] and SSP in a time-efficient manner e.g. two to four 5-min training sessions per week [20]. Further, PJT may induce improvements in neuromuscular, cardiovascular and body composition-related measures of physical fitness and SSP [20, 29–33]. Moreover, PJT is safe for youths and a key element of injury prevention programs [34].

However, most PJT studies have been conducted with adults, with comparatively fewer studies in youths [35, 36]. The transfer of findings from adults to youths appears inappropriate considering that during the youths’ maturation process a cascade of biological events leads to e.g., rapid increases in stature, potential temporary disruption in motor co-ordination, large increases in fat-free mass, changes in muscle–tendon architecture [37–39], all of which may influence the physical fitness and SSP responsiveness to PJT [37, 40–48]. More specifically, the way maturation influences the PJT response on physical fitness and SSP remains unresolved [37, 48–54]. Those PJT studies that reported information on maturity have used different methods to estimate participants’ maturity status (e.g., age at peak height velocity [PHV]; Tanner stages) [35, 36]. This makes a comparison between studies difficult. Although there is evidence on the effects of PJT on physical fitness and SSP in athletes aged 10–18 years [48, 51, 52, 54], comparisons across these age groups are scarce. For example, Asadi et al. [51] noted in their meta-analysis, which included 16 PJT studies with athletes aged 10–18 years, that none of the studies included two or more maturity groups. Due to this limitation, the aforementioned review study [51] compared the results between the 16 PJT studies according to chronological age but not maturity status. Greater performance gains were found for PJT studies with 13.0–18.0 years participants (ES = 0.95 − 0.99) compared to their younger peers (10.0–12.9 years; ES = 0.68) [51]. This analysis however could be confounded due to differences in the applied PJT protocols (e.g., duration; intensity) which may affect the results of PJT studies [51]. Further, chronological age-based comparisons fail to account for the variation in maturity status within the age groups. Moreover, another systematic review [54] compared the effects of PJT in different maturity groups (estimated using the reported chronological age) and, in contrast to Asadi and colleagues [51], found greater performance gains for younger compared with older participants. It must be noted though that Asadi and colleagues [51] focused their analysis on change-of-direction (COD) speed, while Peitz and colleagues [54] examined a broader spectrum of physical fitness and SSP outcomes. Further, Peitz and colleagues [54] did not provide a meta-analysis but a systematic review with comparative studies. Therefore, there is a need for a meta-analysis to compute outcome-specific results according to the maturity status of the participants involved in PJT.

A systematic review of the currently available literature could be valuable because it may shed light on the effectiveness of PJT on physical fitness and SSP as per the participants’ maturity status, with implications also for health [55, 56]. Additionally, most studies have included relatively small sample sizes (i.e., N < 10) [35, 36], which is a common issue in the sport-science literature [57]. Moreover, most studies related to the effectiveness of PJT on physical fitness and SSP in participants according to their maturity status have not addressed the potential role of moderators such as total *volume* (i.e., minimal effective training dosage), or participants’ sex (i.e., included only males) [35, 36, 51]. Therefore, this systematic review with meta-analysis set out to analyse the body of peer-reviewed articles assessing the effects of PJT on measures of physical fitness and SSP according to the participants’ maturity status.

## Methods

### Procedures

A systematic review was conducted according to international standards [58], and adapted a posteriori according to the updated PRISMA 2020 guidelines [59] and related advances in the field [35, 36, 60–65] (adaptations described in the Additional file 4: Table S1).

### Literature Search: Administration and Update

With no time-frame restrictions, we performed electronic searches in the following databases PubMed, Web of Science, and SCOPUS. A detailed description of our search can be found in the Additional file 4: Table S1. Studies were eligible to be included from inception up to January 2022. One author (RRC) conducted the initial search and removed duplicates.

In accordance with the a priori defined study eligibility criteria (Table 1), two authors (RRC and FMC) independently screened and confirmed, the retrieved studies based on titles, abstracts, and full texts. A third author (UG) was consulted in case of disagreement between RRC and FMC. The respective case was discussed until we reached a unanimous decision. Potentially relevant studies were also searched for in the reference lists of the included studies.

**Table 1:** Selection criteria used in the meta-analysis

Category	Inclusion criteria	Exclusion criteria
Population	Youth* participants aged < 18 years (i.e., group mean value), with no restrictions on their physical fitness or sport-specific performance level, sport practiced, competitive level, or sex	Participants with health problems (e.g., injuries, recent surgery), precluding participation in a plyometric-jump training program
Intervention	A plyometric-jump training program lasting ≥ 2 weeks with ≥ 6 total training sessions, which included unilateral and/or bilateral jumps, loaded or unloaded exercises, with repeated (cyclical) or non-repeated (non-cyclical) jumps, which commonly utilize long (countermovement jump) or short (drop jump) stretch shortening cycle	Exercise interventions not involving plyometric-jump training (e.g., upper-body plyometrics only training interventions) or exercise interventions involving plyometric-jump training programs representing less than 50% of the total dedicated-intervention training load (i.e., lower-limbs number of exercises) when delivered in conjunction with other training interventions (e.g., high-load resistance training)
Comparator	Studies comparing groups of different maturity status with or without active or passive control groups. According to standardised methods, comparisons included well-defined maturation groups (e.g., Tanner stages; estimation of age at peak height velocity; x-ray methods [e.g., Fels method])	Absence of a well-matched maturation-age comparator group (i.e., studies including different maturation-age groups that also differ in other relevant moderators [e.g., physical fitness or sport-specific performance level; sex]) Studies comparing different maturation-age groups but exposed to different plyometric-jump training interventions
Outcome	At least one measure related to physical fitness (e.g., jump height) or sport-specific performance (e.g., soccer ball kicking velocity) before and after the training intervention	Lack of baseline and/or follow-up data
Study design	Experimental design with a control group, and two or more maturity groups exposed to the same plyometric jump-training stimulus (e.g., type of exercises, intensity, *volume*, frequency)	Single-group interventions

*The conceptual and operational definition for the term’s “youth”, “children”, “adolescent”, “young”, “toddler” (among others) is very plastic in the literature. Therefore, the umbrella term “youth” (< 18 years) was consistently preferred in our manuscript

Included studies and selection criteria were sent to three experts related to the research area PJT to help identify additional relevant articles (the experts did not receive the search strategy). Thereafter, included studies were assessed for errata or retraction.

### Inclusion and Exclusion Criteria

Selection of studies was based on the PICOS approach [58]. Although most PJT studies are published in English [35], we included studies written in English, Spanish, Portuguese, and German (i.e., authors’ native languages). Only original full-text peer-reviewed articles were included, excluding other documents (e.g., books, book chapters, congress abstracts).

Regarding the comparison between groups of different maturity status, various methods were identified that estimate the individual’s maturity status such as Tanner stages [66], anthropometric characteristics to estimate PHV or similar somatic maturity prediction models [67–69], X-ray methods [70], or related methods (e.g., pre- and post-menarche) [71]. Nonetheless, it is difficult to compare all identified methods to estimate the maturity status. Although we did not exclude studies a priori that used different methods to determine the maturity status, we preferred those approaches that are easy-to-administer for practitioners (e.g., PHV through anthropometric characteristics). In addition, previous PJT studies indicated that PHV and Tanner stages are the two most common methods used by researchers and practitioners [35, 36]. Studies reporting only chronological age or competitive age categories were excluded.

### Data Extraction

In this study, we evaluated the effects of PJT (compared to maturity-matched controls and between maturity groups) on different measures of physical fitness and SSP (see below), as these may reflect different physiological and biomechanical markers related to the health status, the injury risk, and sport-related long-term effective participation [2, 72–75]. Tests that assess physical fitness attributes such as jump performance (e.g., countermovement jump [CMJ] height), linear sprint speed (e.g., 20-m sprint time), balance (e.g., one-legged stance time), endurance (e.g., 20-m shuttle run test), and muscle strength (one-repetition maximum of leg extensors) show very high test–retest reliability with an intraclass correlation coefficient of > 0.9 [76–81], which is essential to ensure strong consistency between the analysed studies within a meta-analysis [58].

If one study included two or more tests related to the same outcome category (e.g., 20-m shuttle run test for endurance, and Yo-Yo test for endurance), we included the test that is most representative of the respective outcome variable under consideration. This was based on the following logical hierarchical rationale: (i) greater representativeness compared to other study tests [e.g., if three studies reported the 20-m shuttle run test for endurance, and one additional study reported both the 20-m shuttle run test for endurance, and the Yo-Yo test for endurance, the 20-m shuttle run test was selected]; (ii) greater specificity to the group of participants being compared [e.g., for soccer players, the Yo-Yo test for endurance would be preferred over the 20-m shuttle run test for endurance]; (iii) greater reliability. Further, only data from the final period was included if one study included two or more post-intervention test periods (e.g., 6-week, 12-week). However, if the post-intervention period involved a detraining period, this was not included. Relatedly, if one study included two or more measures from a single test (e.g., vertical jump height and ground contact time from the drop jump test), the above-mentioned logical hierarchical rationale was applied. In addition, in the context of this study, performance measures (e.g., vertical jump height) were of greater interest compared with biomechanical variables (e.g., joint angle at contact with the ground).

The means and standard deviations of dependent variables were extracted as previously reported [171], using specialised software when studies reported data only in figures [82]. Four authors were independently involved in the process (RRC, FMC, JA, UG).

### Methodological Quality of the Included Studies

The studies’ methodological quality was assessed as previously described [171], using a valid and reliable tool which is the PEDro scale [83–85], frequently used in the PJT literature [36, 80, 86]. However, it is not possible to satisfy all scale items in PJT studies [87]. Specifically, for the present systematic review, (i) for PEDro scale item number 2, a point was awarded when participants were divided according to age/maturity, (ii) for PEDro scale item number 3 concealed allocation was precluded, as participants were allocated to groups according to their age/maturity; therefore, this item was not considered in the final classification of the study quality; (iii) for PEDro scale item number 4, baseline differences were expected due to differences in age/maturity [71]; therefore, this item was not considered in the final classification of the study quality. Therefore, PJT studies were assessed as previously recommended [26, 86, 88]: “poor”, “moderate”, and “high” quality for ≤ 3 points, 4–5 points, and 6–10 points, respectively. Two authors applied the PEDro scale for each included study independently (RRC and FMC). In cases when an author encountered studies of which he was a co-author, his assessment was not considered, and any discrepancies between authors were resolved via consensus or with the assistance of a third author (UG). We did not exclude studies if they had low methodological quality; however, moderator analyses according to the studies’ methodological quality were planned (see below, “*Moderator analyses*” section).

### Summary Measures, Synthesis of Results, and Publication Bias

According to Valentine and colleagues, two studies are needed to aggregate and meta-analyse results [89]. However, low sample sizes are common in the field of PJT [17, 35, 36, 57, 90]. Therefore, we performed meta-analyses when ≥ 3 studies were available [2, 91]. The ES (i.e., Hedges’ *g*, with 95% confidence intervals [95% CIs]) for each physical fitness and SSP attribute in the compared groups (i.e., maturity-matched PJT group vs. control group; PJT group vs. PJT group with different maturity) was calculated using the DerSimonian and Laird inverse random-effects model for meta-analyses. Calculated ES were interpreted as trivial, small, moderate, large, very large, and extremely large for values < 0.2, 0.2–0.6, > 0.6–1.2, > 1.2–2.0, > 2.0–4.0, and > 4.0, respectively [92]. The *I*^2^ statistic values of < 25%, 25 − 75%, and > 75% represented low, moderate, and high levels of heterogeneity, respectively [93]. The Comprehensive Meta-Analysis software (version 2, Biostat, Englewood, NJ, USA) was used for statistical analyses (significance set at *p* ≤ 0.05).

#### Assessment of Maturity Status

Most included PJT studies reported the maturity status according to years from PHV [35, 36]. Although PHV is not the gold standard, it is valid and logistically convenient for practitioners [56, 71, 94]. Therefore, we used a proxy of PHV as a basis to pool maturity judged by different methods. To avoid the exclusion of potentially relevant studies which reported pubertal staging (i.e., Tanner stages), PHV was obtained from sex-specific equations due to its relationship to the aforementioned maturity-age markers [95]. We dichotomised PHV categorization, as previously suggested [67]. Therefore, any negative maturity offset (e.g., PHV − 0.5) classified the group mean as pre-PHV and any positive maturity offset as post-PHV. For dichotomization of groups in studies that reported the maturity status according to the Tanner method, stages I–III for boys and I–II for girls were considered as pre-PHV (i.e., < 50% of the corresponding sex-group achieved PHV for the respective Tanner stage). Stages IV–V for boys and III–V for girls were deemed as post-PHV (i.e., > 50% of the corresponding sex-group achieved PHV for the respective Tanner stage) [95]. However, when authors self-reported dichotomization (e.g., pre-, and post-menarche), this was used for further analysis. Of note, a priori, we considered maturity categorization for comparisons as pre-PHV (i.e., < − 1.0 y PHV), mid-PHV (i.e., -1.0 to 1.0 y PHV), and post-PHV (i.e., > 1.0 y PHV) [48, 51, 67]. However, a posteriori, the insufficient number of studies per outcome and maturity category precluded the categorization.

### Moderator Analyses

#### Subgroup Analyses

The participant’s sex was considered a potential moderator variable because, in addition to the well-known sex-related differences in maturation-related biological processes [66], physical fitness and SSP adaptive responses to PJT programmes may be affected by the participant’s sex [96].

#### Single Training Factor Analysis

Single training factor analyses were computed for the programme duration (number of weeks and total number of training sessions) [96] and training frequency (number of sessions per week) [97], based on the reported influence of these variables on physical fitness and SSP adaptations following PJT. Additional moderators such as total number of jumps, and type of jump (i.e., unilateral, bilateral, mixed) were also considered if the studies provided such data. Further, as combined training (e.g., PJT combined with traditional controlled-velocity resistance training) may induce different outcomes in comparison to PJT or traditional resistance training in isolation in youth, particularly at different stages of maturity [98], moderator analyses were performed to compare results from studies that delivered PJT only or in conjunction with other training interventions. Given the challenges of determining/quantifying PJT intensity (and the poor reporting of this factor in the literature), this was not considered for the single training factor analysis.

The median split technique was applied as previously recommended and described [99–101] for moderator analyses.

#### Study Methodological Quality

Comparisons of results between studies with ≤ 3 points, 4–5 points, and 6–10 points on the PEDro scale were planned.

### Additional Analyses

#### Certainty of Evidence

The GRADE rating system was applied by two authors (JA and RRC), according to previously published criteria [102–106].

## Results

### Study Selection

**Fig. 1:**
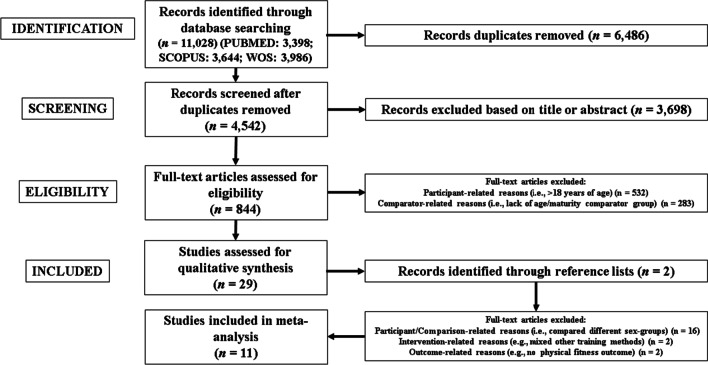
Flow chart illustrating the study selection process

Twenty-nine studies were eligible for qualitative synthesis. Two additional studies were identified through reference list screening [107, 108]. Another 20 studies were discarded (for reasons detailed, see Additional file 5: Table S2). Finally, eleven studies were included in this meta-analysis [109–119] Fig. 1 illustrates the search process and study selection).

### Methodological Appraisal of the Included Studies

The eleven included studies had a median (i.e., nonparametric) PEDro score of 5 points. More details can be found in Table 2.

**Table 2:** Methodological quality of the included studies using the PEDro rating scale

Study N°	1	2^¶^	3^§^	4^¥^	5	6	7	8	9	10	11	Score*	Study quality
Asadi et al. [109]	1	1	0	0	0	0	0	1	1	1	1	5	Moderate
Davies et al. [110]	1	1	0	0	0	0	0	1	1	1	1	5	Moderate
Heinonen et al. [111]	1	1	0	0	0	0	0	1	1	1	1	5	Moderate
Lloyd et al. [112]	1	1	0	0	0	0	0	1	1	1	1	5	Moderate
Lloyd et al. [113]	1	1	0	0	0	0	0	1	1	1	1	5	Moderate
Moran et al. [114]	1	1	0	0	0	0	0	1	1	1	1	5	Moderate
Ramirez-Campillo et al. [115]	1	1	0	0	0	0	1	1	1	1	1	6	High
Romero et al. [116]	1	1	0	0	0	0	1	1	1	1	1	6	High
Uzelac-Sciran et al. [117]	1	1	0	0	0	0	0	1	1	1	1	5	Moderate
Vera-Assaoka et al. [118]	1	1	0	0	0	0	1	1	1	1	1	6	High
Vilela et al. [119]	1	1	0	0	0	0	0	1	1	1	1	5	Moderate

A detailed explanation for each PEDro scale item can be accessed at https://www.pedro.org.au/english/downloads/pedro-scale.

^*^From a possible maximal score of 10

^¶^In the context of this systematic review, a point was awarded when participants were divided according to age/maturity

^**§**^Concealed allocation was precluded, as participants were allocated to groups according to their age/maturity. Therefore, this item was not considered in the final classification of the study quality

^¥^Differences were expected due to differences in age/maturity. Therefore, this item was not considered in the final classification of the study quality

### Study Characteristics

Tables 3 and 4 provide a description of the study participant characteristics and PJT interventions. More information on the control groups and whether active or passive can be found in Additional file 2.

**Table 3:** Participants’ characteristics

	Sex	Years of age (maturity)	Body mass (kg)	Height (cm)	SPT	Sport*	Season period
Asadi et al. [109]	Male	11.5 ( − 1.8 y PHV)	31.0	138.3	No	Soccer	Pre-season
		14.0 (0.3 y PHV)	43.5	154.5			
Davies et al. [110]	Female	11.7 (0.2 y PHV)	42.9	154.0	No	NA	NA
		14.3 (2.5 y PHV)	59.9	166.3			
Heinonen et al. [111]	Female	11.7 (Tanner I-II)	39.3	147.9	NR	NA	NA
		13.7 (Tanner IV-V)	52.5	161.2			
Lloyd et al. [112]	Male	12.7 ( − 1.5 y PHV)	56.0	159.6	No	NA	A
		16.4 (1.3 y PHV)	67.8	179.5			
Lloyd et al. [113]	Male	12.3 ( − 1.9 y PHV)	44.8	151.9	No	NA	NA
		15.3 (1.1 y PHV)	65.0	174.4			
Moran et al. [114]	Male	12.6 ( − 1.5 y PHV)	50.9	155.4	No	Field hockey	In-season
		14.3 (0.3 y PHV)	58.8	173.1			
Ramirez-Campillo et al. [115]	Male	10.9 ( − 2.3 y PHV)	42.5	148.0	No	Soccer	In-season
		11.2 ( − 2.2 y PHV)	40.8	149.0			
		15.0 (1.7 y PHV)	63.6	169.0			
		15.6 (1.8 y PHV)	63.6	169.0			
Romero et al. [116]	Female	12.7 (Tanner II-IV)	40.9	145.8	No	NA	NA
		16.3 (Tanner IV-V)	54.0	153.9			
Uzelac-Sciran et al. [117]	Male	13.1 ( − 1.0 y PHV)	49.4	159.8	No	NA	NA
		14.0 (0.8 y PHV)	68.5	176.3			
Vera-Asaoka et al. [118]	Male	11.2 (Tanner I-III)	36.8	143.0	No	Soccer	In-season
		14.4 (Tanner IV)	54.7	163.0			
Vilela et al. [119]	Female	10.5 (Tanner II)	38.6	150.0	No	Volleyball	Pre-season
		11.8 (Tanner III)	46.3	160.0			

Abbreviations: ordered alphabetically

*In the studies with participants involved in sports, the control groups involved sport-specific active controls

*y PHV*: years to/from age of peak height velocity; *NA*: not applicable. In the studies indicated as NA, control groups involved school-based physical education active controls; *NR*: not reported; *SPT*: systematic plyometric-jump training

**Table 4:** Characteristics of the plyometric-jump training (PJT) interventions

	Freq	Dur	Int	BH	NTJ	Tply	Comb	RBS	RBR	RBTS	Tsurf	PO	Repl	Taper
Asadi et al. [109]	2	6	Max	20–60	720 (120)*	DJ	No	120	7	72–96	Grass	No	No	No
Davies et al. [110]	2	7	Mix	NA	2,204 (315)	Mix	No	5–60	NA	≥ 48	NR	T, V	Yes	Yes
Heinonen et al. [111]	1.3	36	NR	30	10,800 (300)	Mix	Step-aerobic	NR	NR	NR	NR	T, V	No	No
Lloyd et al. [112]	2	6	NR	20	958 (160)	Mix	No	60–120	NR	> 48	NR	T, V	NA	No
Lloyd et al. [113]	2	4	Max	20	740 (185)	Mix	No	~ 90	NR	≥ 48	NR	T, V	Yes	No
Moran et al. [114]	2	6.5	Max	NA	600 (92)	Mix	No	NR	NA	NR	NR	T	Yes	No
Ramirez-Campillo et al. [115]	2	6	Max	Optimal	1,008 (168)	Mix	No	30 vs 120	3	≥ 48	Mix	V	Yes	Yes
Romero et al. [116]	2	6	Max	5–35	1,590 (265)	Mix	No	30–60	5–15	72–96	Mix	V	Yes	Yes
Uzelac-Sciran et al. [117]	2	8	NR	NR	1,336 (167)	Mix	No	60–120	NR	48	Parquet	T, V	NR	No
Vera-Assaoka et al. [118]	2	7	Max	20–60	840 (120)	DJ	No	90	15	≥ 48	Grass	No	Yes	No
Vilela et al. [119]	3	8	Mix	20–60	3,510 (439)	Mix	No	60–120	NA	48–72	Wood	Int, T, V	No	Yes

Abbreviations ordered alphabetically: *BH*: box or obstacle height (cm) used in PJT exercises; *COD*: change of direction drills; *Comb*: combined PJT exercises with other type of exercise (e.g., other than regular training practice for those who were athletes); *DJ*: drop jump; Duration: total duration (weeks) of PJT interventions; *Freq*: frequency of PJT training (sessions per week); *Int*: intensity of PJT intervention exercises; *Max*: maximal; *NA*: not applicable; *NTJ*: number of total jumps (e.g., foot contacts per leg). In some studies, jump repetitions were prescribed as distance or time. For this review, 1-m and 1-s were considered equivalent to 1 repetition; *NR*: not reported; *PO*: progressive overload (either as intensity, type of exercise, volume, or a combination of these); *RBR*: rest between repetitions (seconds); *RBS*: rest between sets (seconds); *RBTS*: rest between PJT sessions (hours); *Repl*: replaced part of their regular activities (e.g., warm-up before a regular soccer training session) with PJT. If “No”, this implies that participants added PJT to their regular training schedule; *RT*: resistance training (e.g., slow-speed heavy-load squat); *T*: technique or type of exercise; *Tply*: type of PJT exercises; *Tsurf*: type of surface during PJT sessions; *V*: volume (i.e., number) of jumps; *VS*: two or more groups were compared (with the same maturity), using different PJT programming variables

*Values in parenthesis denote the number of jumps per week

Briefly, participants involved in PJT totalled 367 (28 groups), and control participants totalled 377 (28 groups). Seven studies recruited male participants and four studies recruited female participants (Table 3). Seven studies reported maturity using PHV and four studies using Tanner staging (Table 3). Between one and three weekly exercise sessions were applied in the PJT interventions. The exercise programmes lasted between 4 and 36 weeks. Most studies (*n* = 10) had an exercise period of no more than 8 weeks, with a median duration of ~ 7 weeks. The PJT intensity (Table 4) was reported in eight studies, through different indices (e.g., reactive strength index [RSI]; height/distance; jump technique [e.g., from two-leg to one-leg; landing technique]).

### Meta-analyses

Table 5 and Fig. 2 summarise the main values derived from the meta-analyses. A detailed presentation of the meta-analyses per outcome, including figures, 95% CI values, I^2^ statistics, and other results, is provided in the Additional file 1: Figures S1-S24.

**Table 5:** Effect size and p-values derived from meta-analyses

	PJT pre-PHV versus control pre-PHV	PJT post-PHV versus control post-PHV	PJT pre-PHV versus PJT post-PHV
*Sport-specific performance*^*§*^* (3:0)*^*¥*^	0.55 (**0.004**)*	0.82 (< **0.001**)*	0.11 (0.565)
Male studies	Same as above	Same as above	Same as above
Female studies	NA	NA	NA
*Maximal dynamic strength (2:2)*	0.35 (**0.022**)	0.46 (**0.003**)	-0.09 (0.546)
Male studies	NA	NA	NA
Female studies	NA	NA	NA
*COD speed time (1:3)*	0.62 (0.075)	0.51 (0.149)	-0.42 (**0.012**)^¶^
Male studies	NA	NA	NA
Female studies	0.37 (0.29)	0.48 (0.366)	-0.40 (0.099)
*Linear sprinting speed time (5:1)*	0.38 (**0.011**)	0.50 (**0.002**)	0.00 (0.980)
Male studies	0.34 (**0.034**)	0.39 (**0.017**)	-0.05 (0.739)
Female studies	NA	NA	NA
*Horizontal jump distance (3:1)*	0.42 (**0.006**)	0.56 (< **0.001**)	0.08 (0.583)
Male studies	0.32 (0.083)	0.59 **(0.001)**	0.20 (0.285)
Female studies	NA	NA	NA
*Squat jump height (2:2)*	0.46 (**0.011**)	0.20 (0.291)	-0.13 (0.558)
Male studies	NA	NA	NA
Female studies	NA	NA	NA
*Reactive strength index (5:2)*	0.57 (< **0.001**)	0.40 (**0.002**)	-0.12 (0.330)
Male studies	0.66 (< **0.001**)	0.45 (**0.001**)	-0.11 (0.416)
Female studies	NA	NA	NA
*Countermovement jump height (4:3)*	0.50 (0.088)	0.36 (0.061)	0.10 (0.499)
Male studies	0.87 (0.084)	0.51 (0.090)	0.03 (0.878)
Female studies	0.12 (0.637)	0.16 (0.501)	0.27 (0.462)

*Values denote effect size (p-value; bold values denote significance)

^¶^Favouring the pre-PHV group

^§^Studies involved sport-specific testing for ball kicking velocity/distance, except one study that involved dribbling velocity. However, when removed from the analysis, the results remained consistent across comparisons (details in Additional file 1: Figs. S1, S9 and S17)

^¥^Denotes the number of studies included in meta-analysis conducted in males:females

*NA*: not available analysis, due to lack of adequate number (i.e. ≤ 3) of studies for meta-analysis; *PHV*: age of peak height velocity; *COD*: change-of-direction; *PJT*: plyometric-jump training

**Fig. 2:**
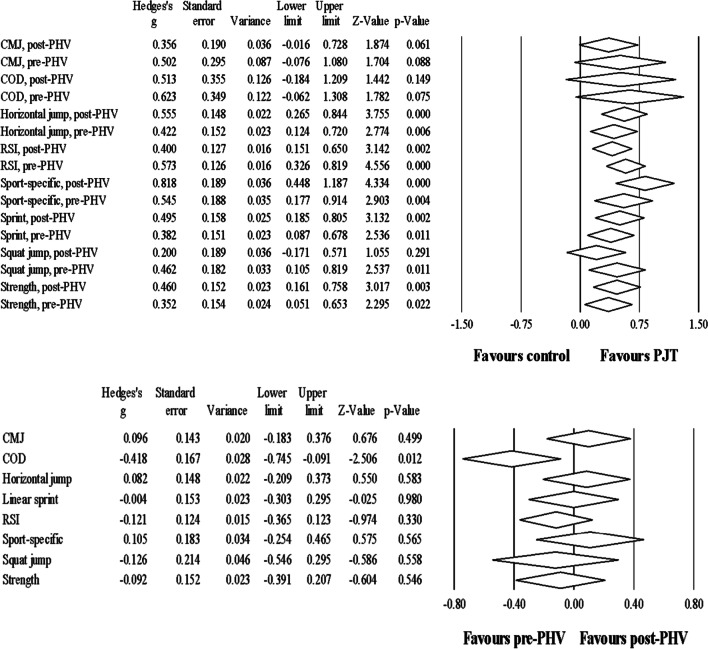
Upper figure: changes in physical fitness and sport-specific performance outcomes after PJT compared to controls in both pre-PHV and post-PHV participants. Lower figure: changes in outcomes after PJT in pre-PHV compared to post-PHV participants. *CMJ*: countermovement jump. *COD*: change of direction speed time. *PHV*: peak height velocity. *PJT*: plyometric jump training. *RSI*: reactive strength index

When compared to controls, both pre-PHV and post-PHV participants obtained small-moderate improvements (ES = 0.35 − 0.80) in most outcomes (i.e., SSP [soccer ball kicking velocity/distance; soccer ball dribbling velocity]; maximal dynamic strength; linear sprint; horizontal jump; RSI) after PJT. However, COD speed (ES = 0.51 − 0.62, *p* = 0.149–0.075) and CMJ height (ES = 0.36 − 0.50,* p* = 0.088 − 0.061) were not significantly improved in both pre-PHV and post-PHV participants after PJT when compared to controls. The SJ height improved after PJT to a small magnitude in both pre-PHV and post-PHV participants compared to controls (ES = 0.46 − 0.20, respectively), although only significantly in the former.

When pre-PHV and post-PHV participants involved in PJT were compared, no significant differences were noted between maturity groups regarding changes in most physical fitness and SSP outcomes, except COD speed (small ES = -0.42, favouring pre-PHV participants).

#### Meta-analyses According to Moderators

The Additional file 3 provides a full description of meta-analyses according to moderators. Briefly, no significant effects were noted for any moderator, including participants’ sex, PJT programme duration (number of weeks and total number of training sessions), PJT programme total number of jumps, and studies' methodological quality.

### Certainty of Evidence

The GRADE analyses are provided in Table 6. According to the GRADE assessments, the certainty of evidence was considered low to very low across outcomes and groups comparisons.

**Table 6:** GRADE analyses

	Studies and PSS	Risk of bias in studies	Inconsistency	Imprecision	Certainty of evidence
Sport-specific performance					
Pre-PHV participants: PJT versus controls	3, *n* = 108	Some concerns	Low	Small effect favouring PJT	⊕ ⊕ , Low
Post-PHV participants: PJT versus controls	3, *n* = 112	Some concerns	Low	Moderate effect favouring PJT	⊕ ⊕ , Low
PJT Pre-PHV versus PJT Post-PHV participants	3, *n* = 110	Some concerns	Low	No clear direction of effects	⊕ , Very low
Horizontal jump distance					
Pre-PHV participants: PJT versus controls	4, *n* = 166	Some concerns	Low	Small effect favouring PJT	⊕ ⊕ , Low
Post-PHV participants: PJT versus controls	4, *n* = 180	Some concerns	Low	Small effect favouring PJT	⊕ ⊕ , Low
PJT Pre-PHV versus PJT Post-PHV participants	4, *n* = 174	Some concerns	Low	No clear direction of effects	⊕ , Very low
Maximal dynamic strength					
Pre-PHV participants: PJT versus controls	4, *n* = 168	Some concerns	Low	Small effect favouring PJT	⊕ ⊕ , Low
Post-PHV participants: PJT versus controls	4, *n* = 173	Some concerns	Low	Small effect favouring PJT	⊕ ⊕ , Low
PJT Pre-PHV versus PJT Post-PHV participants	4, *n* = 171	Some concerns	Low	No clear direction of effects	⊕ , Very low
Change of direction speed time					
Pre-PHV participants: PJT versus controls	4, *n* = 132	Some concerns	Moderate	No clear direction of effects	⊕ , Very low
Post-PHV participants: PJT versus controls	4, *n* = 152	Some concerns	Moderate	No clear direction of effects	⊕ , Very low
PJT Pre-PHV versus PJT Post-PHV participants	4, *n* = 146	Some concerns	Low	Small effect favouring pre-PHV	⊕ ⊕ , Low
Linear sprinting speed time					
Pre-PHV participants: PJT versus controls	6, *n* = 171	Some concerns	Low	Small effect favouring PJT	⊕ ⊕ , Low
Post-PHV participants: PJT versus controls	6, *n* = 162	Some concerns	Low	Small effect favouring PJT	⊕ ⊕ , Low
PJT Pre-PHV versus PJT Post-PHV participants	6, *n* = 164	Some concerns	Low	No clear direction of effects	⊕ , Very low
Squat jump height					
Pre-PHV participants: PJT versus controls	4, *n* = 119	Some concerns	Low	Small effect favouring PJT	⊕ ⊕ , Low
Post-PHV participants: PJT versus controls	4, *n* = 110	Some concerns	Low	No clear direction of effects	⊕ , Very low
PJT Pre-PHV versus PJT Post-PHV participants	4, *n* = 109	Some concerns	Low	No clear direction of effects	⊕ , Very low
Reactive strength index					
Pre-PHV participants: PJT versus controls	7, *n* = 252	Some concerns	Low	Small effect favouring PJT	⊕ ⊕ , Low
Post-PHV participants: PJT versus controls	7, *n* = 238	Some concerns	Low	Small effect favouring PJT	⊕ ⊕ , Low
PJT Pre-PHV versus PJT Post-PHV participants	7, *n* = 246	Some concerns	Low	No clear direction of effects	⊕ , Very low
Countermovement jump height					
Pre-PHV participants: PJT versus controls	7, *n* = 195	Some concerns	Moderate	No clear direction of effects	⊕ , Very low
Post-PHV participants: PJT versus controls	7, *n* = 192	Some concerns	Moderate	No clear direction of effects	⊕ , Very low
PJT Pre-PHV versus PJT Post-PHV participants	7, *n* = 189	Some concerns	Low	No clear direction of effects	⊕ , Very low

(i) *Risk of bias in studies*: judgments were downgraded by one level (i.e., some concerns) if the average PEDro scores were moderate (< 6) or by two levels (i.e., high risk) if they were poor (< 4); (ii) *Indirectness*: low due to eligibility criteria (not featured in the table); (iii) *Risk of publication bias*: not assessed because all comparisons derived from less than 10 studies; (iv) *Inconsistency*: judgments were downgraded by one level when the impact of statistical heterogeneity (*I*^2^) was high (> 75%); (v) *Imprecision*: one level of downgrading occurred whenever < 800 participants were available for a comparison and/or if there was no clear direction of the effects. When both were observed, certainty was downgraded by two levels

*GRADE*: grading of recommendations assessment, development and Evaluation; *PSS*: pooled sample size

## Discussion

This systematic review with meta-analysis set out to analyse the body of peer-reviewed articles assessing the effects of PJT on physical fitness and SSP outcomes according to participants’ maturity status. Overall, the between-maturity group comparisons were non-significant, except for COD. A discussion is provided regarding current findings, including the potential role of incidental, casual, and statistical artefacts, as well as potential deeper mechanisms that should be explored in future studies, and the role of potential moderators. Indeed, the non-significant role of maturity on physical fitness and SSP adaptation after PJT is somewhat surprising. Indeed, most of the original studies included in our systematic review [109–119] and comparative studies [37, 48, 50–52, 54] found that maturity moderated PJT effects. Moreover, it is worth noting that the range of improvement for the different physical fitness and SSP outcomes after PJT in the pre-PHV group was relatively reduced with ES values from 0.35 (maximal dynamic strength) to 0.61 (COD speed time). In comparison, the post-PHV group achieved a wider range of ES values from 0.20 (squat jump height) to 0.82 (SSP). A discussion of the main findings is provided in the following sections.

### Maximal Dynamic Strength

In relation to maximal dynamic strength, small significant improvements (ES = 0.35 − 0.46, all *p* < 0.05) were noted in pre-PHV and post-PHV participants after PJT, as compared to control groups. Furthermore, such improvements were similar for both the pre-PHV and post-PHV participants. These observations remained after meta-analyses of relevant moderators of the main effect which included the participants’ sex, the PJT programme duration (number of weeks and total number of training sessions), the total number of jumps in the programme and methodological quality of the studies. It was unsurprising that only small improvements were seen in both the pre-PHV and post-PHV groups when one considers the principle of specific adaptation to imposed demands in relation to training. It is likely that for larger increases in maximal dynamic strength to occur, the study participants would need to have been exposed to more appropriate exercises to achieve that outcome such as traditional resistance training with higher external loads. Indeed, a previous meta-analysis [120] reported a trivial effect (ES = 0.16) and a large effect (ES = 1.14) for lower body strength in youth participants after power training and strength training, respectively.

Although muscular strength might improve in line with advancing age and maturity, the specific effects of puberty may not always be noticed in the short-term. This could occur because there is a mismatch between the meaningful rate of physical changes and the time that the neural system requires to adapt to them [38, 66, 71]. Further, most studies in our meta-analyses had a duration of < 12 weeks. During such a short timeframe, physiological adaptations can most likely be attributed to neuromuscular improvements (e.g., motor unit recruitment and firing rate) [25, 121]. However, longer-term training interventions (> 12 weeks) may increase the relative contribution of muscle hypertrophy to training-related adaptations [121], thus inducing greater strength gains over longer training periods [122]. Older youths may exhibit a better hormonal milieu (e.g., higher testosterone levels) for muscle hypertrophy to occur as compared to younger youths who lack this characteristic [38]. As PJT can exert a considerable hypertrophic effect [123, 124], greater strength improvements, as a result of a higher potential for muscular hypertrophy, in post-PHV as compared to pre-PHV participants might be expected in the longer-term (e.g., > 12 weeks). Despite this, amongst the studies we meta-analysed, one PJT intervention of 36 weeks [111] produced no significant differences in the maximal dynamic strength of the leg extensors between pre-PHV and post-PHV participants. The cited study [111] included female participants only, a population which may be less likely to exhibit hypertrophic responses to training [101]. Accordingly, it remains unclear whether PJT can induce greater strength improvements (via greater hypertrophy) in post-PHV as compared to pre-PHV male youths. Future studies must incorporate more robust analyses particularly considering the low to very low certainty of evidence (Table 6) for maximal strength changes after PJT in both pre-PHV and post-PHV participants.

### Jump-Related Performance

In relation to jump-related measures, when compared to their maturity-matched control group counterparts, pre-PHV participants had larger gains in RSI than post-PHV participants, particularly in males (ES = moderate vs. small; albeit non-significant). This appears to match with the synergistic adaptation theory which suggests that training adaptations are amplified in line with the similar physiological changes that occur in the body during growth and maturation [37, 112, 125]. Of particular importance is muscular fitness, which is an umbrella term for muscle strength, muscle power and muscle endurance [126]. Indeed, youths in pre-PHV can experience an accelerated development of explosive strength (e.g., rate of force development) and muscular power during a period that may be optimal for increasing muscular fitness through training [48, 127]. The horizontal jump movement requires greater absolute force production for execution and likely targets a different expression of the stretch–shortening cycle (SSC) when compared to the RSI, which might favour the nature of the type of adaptations experienced by post-PHV participants exposed to PJT [37, 112, 125]. Indeed, albeit a non-significant difference was noted for the effect of PJT on the horizontal jump performance change in male post-PHV compared to pre-PHV youths (ES = 0.20; *p* = 0.285), male post-PHV youths attained a greater (ES = 0.59) horizontal jump improvement when compared to their pre-PHV counterparts (ES = 0.32).

Of note, the SJ improved significantly when compared to control subjects only in pre-PHV group (ES = 0.46), a result that might be related to the low specificity between PJT exercises (i.e., involving the SSC) and SJ (i.e., shortening muscle action). Although this is true for both pre-PHV and post-PHV groups, the low training specificity (e.g., type of jump; reduced inter-set recovery) may affect post-PHV participants’ muscular fitness to a larger extent [37, 43, 115]. For example, pre-PHV participants may obtain a greater concentric stimulus from PJT than post-PHV participants because of differences in muscle activation patterns [128]. Indeed, post-PHV participants can exhibit a more excitatory muscle activation pattern during PJT activities, and thus more activity will arise from pre-activation and stretch reflexes, meaning greater contribution from eccentric activation [128]. Pre-PHV participants will have a more inhibitory response of the aforementioned mechanisms, and thus be more reliant on muscle activation during the propulsive/concentric phase [128]. Indeed, among the analysed outcomes in our meta-analyses, the SJ showed the lowest magnitude in terms of performance improvement after PJT in post-PHV participants while it was the fifth lowest for the pre-PHV participants. However, the extent to which youth participants (and most particularly those in post-PHV) are sensitive to the transference effect [27, 129–134] of PJT activities to a given testing exercise (e.g., concentric-only vs. SSC-based; vertical vs. horizontal; unilateral vs. bilateral), remains to be elucidated. From a practical point of view, although specificity of training is a key element of adaptation [120], a combination with non-specific PJT exercises may offer greater adaptations [27, 134, 135], particularly for athletes already performing specific jumps in the main training session. Indeed, merely replicating those jumps would repeat the same training stimulus, potentially contributing to overuse injuries [136–138].

For CMJ, the magnitude of change was in favour of the PJT groups compared with controls in pre-PHV (ES = 0.50, *p* = 0.088) and post-PHV participants (ES = 0.36, *p* = 0.061). However, neither result achieved the level of statistical significance. Whether the experimental groups were exposed to insufficient PJT doses is unclear, as the minimal effective dose has not been clarified in the literature. However, our moderator analyses revealed no effect of dose, in the form of PJT number of weeks, PJT number of training sessions, or PJT programme total number of jumps. Another possible explanation for the lack of significant differences between PJT participants and their controls is the training level of the controls. The included studies used school-based physical education (*n* = 3) as well as sport-specific activity (*n* = 4) as control conditions. Both control conditions involve habitual engagement in play activities, exercise drills and sports-related activities that mimic the nature of PJT exercises, such as jumps, sprints, and quick changes of direction. Such active-control conditions may have induced physiological adaptations favouring jump performance. At the same time, expected PJT effects compared with these active controls might be diminished. Another potential reason may be related to the sex of the participants. Previous meta-analyses reported a lower magnitude of CMJ performance improvement in females (ES = 0.57) [52] compared to males (ES = 0.73) [48] after PJT interventions. Indeed, in our meta-analyses the PJT-control comparisons indicated that males involved in PJT achieved small-moderate CMJ height improvements (ES = 0.51 − 0.87) compared to their maturity-matched controls. In contrast, the PJT-control comparison for the females involved in PJT revealed a trivial CMJ height improvement (ES = 0.12 − 0.16) compared to their maturity-matched controls. However, the moderator analysis according to participants’ sex indicated that pre-PHV and post-PHV participants responded similarly to PJT in relation to the outcome of CMJ height.

### Sprinting and Sport-Specific Performance

Maximal-intensity short-duration linear and COD running movements frequently occur in youth male and female basketball (every 1–2 s), soccer (every 3–6 s), and handball (every 5–6 s) matches [139, 140], and are common before scoring actions in sports, such as soccer [141]. Muscular strength and power (e.g., jumping), speed, rate of force development, cognitive and technical skills correlate well with linear and COD running performance across sex, age, and sports disciplines [80, 81, 142–156]. Indeed, linear and COD running performance markers may reflect physiological and biomechanical indicators relevant to reducing injury risk and improving SSP [51, 74, 75, 143–146]. Different training methods with the potential to improve linear and COD running performance (e.g., complex training, sprint training) have been reported in the scientific literature [148, 157–162]. However, PJT appears to be one of the most effective exercise types, requiring fast and powerful movements that utilise the SSC. Indeed, PJT has previously resulted in favourable effects on linear and COD running performance [80, 163] and repeated sprint ability with and without COD [35, 36, 164, 165], in line with improvements in the physiological [25, 166] determinants of linear and COD running performance and associated muscular fitness components such as maximal dynamic strength [97] and jumping capability [167]. Our meta-analyses indicate that linear and COD running performance attained up to moderate magnitude of improvement (ES = 0.38 − 0.62) after PJT in both pre-PHV and post-PHV participants, even when control participants were recreationally active or engaged in sport-specific activity. Of note, linear sprinting improved after PJT compared to controls in both pre-PHV and post-PHV. However, COD running performance achieved magnitudes of improvement of 0.62 and 0.51 for pre-PHV and post-PHV participants respectively following PJT compared to controls, but without statistical significance. When compared to control subjects, these groups were involved in three school-based physical education control groups, and one soccer-specific active control group. Therefore, the fact that control participants were active (school-based physical education; sport-specific active) may partially explain the non-significant COD findings in the pre-PHV and post-PHV participants. Alternative explanatory hypotheses, such as the different proportion of studies conducted on males and females for linear sprinting speed time (5:1, respectively) compared to COD speed time (1:3, respectively), or whether (or not) the dosage of PJT applied was sufficient compared to active controls (moderator analysis precluded due to insufficient studies), warrant future assessment.

In relation to maturity status, pre-PHV participants exposed to PJT improved COD significantly more than post-PHV participants. Thus, the early incorporation of PJT into youth training schedules may offer significant advantages. Of note, all studies included in the meta-analysis assessed pre-programmed COD (i.e., movement with a pre-determined course that was known to the participant). However, the effects of PJT on COD with an unpredictable, reactive, and unplanned component should be considered in future studies. Post-PHV athletes, when compared to pre-PHV participants, might be faster in performing reactive-decisional based COD, as they will likely tend to have greater playing experience in line with cognitive development. However, this is speculative, further highlighting the need for additional investigation in the area. The underlying mechanisms for the greater improvement in COD after PJT in pre-PHV as compared to post-PHV participants are unclear, though they could potentially be related to decreases in relative strength owing to increases in the body mass of the more mature participants [168]. On the other hand, some COD tasks incorporate a cognitive element in performance and due to the heightened neural plasticity that can be experienced in pre-PHV participants, this group may be particularly sensitive to the effects of PJT, particularly considering its meaningful effects on the neuromuscular system [25, 169, 170]. It would be relevant to elucidate changes between pre- and post-PHV participants in maximal concentric and eccentric strength and the relationship of these changes with the change in different phases (e.g., acceleration; deceleration; turns to the right-left; joint and trunk angles during COD and risk of injury) of linear and COD running movements, as well as in COD deficit [171]. During growth and maturation, a cascade of biological events lead to rapid increases in stature, potential temporary disruption to motor co-ordination, large increases in fat-free mass and changes to muscle–tendon architecture [37–39], all of which may influence an individual’s responsiveness to PJT. More specifically, the maturity-related effects of PJT on linear and COD running performance and the underlying mechanisms of adaptations require further investigation.

The largest improvement among the meta-analysed outcomes in the post-PHV group was noted for SSP (ES = 0.82) and the third highest in the pre-PHV group (ES = 0.55). Because participants were taking part in PJT while also taking part in their normal sports training (i.e., they were training both PJT and sport specific skills), this partially supports the hypothesis related to the meaningful transference of PJT-related adaptive effects to athletic performance [27, 129–134, 172], particularly in post-PHV participants. Most of the studies that assessed SSP tested ball kicking velocity/distance with the one exception evaluating dribbling velocity [51]. However, when removed from the analysis, the results remained consistent across comparisons (details in Additional file 1: Fig. S1, S9 and S17). Of note, SSP data could be derived only from male soccer players. Therefore, it is unclear how PJT and maturity might interact to impact on tests that are specific to other sports or amongst female youths.

### Meta-analyses According to Moderators

#### Sex

In a previous study, similar physical fitness and SSP improvements were noted after PJT in adult male and female participants [173]. However, the findings in adult populations appear inapplicable to youths because the adaptive potential in the latter is different due to the timing and tempo of maturational changes that occur during puberty [37, 41–48]. Indeed, when CMJ was assessed, males attained an ES of 0.87 (pre-PHV) and 0.51 (post-PHV) as compared to their maturity-matched controls, while females attained ES values of just 0.12 and 0.16, respectively. Further, it is notable that the main number of total jumps performed by females among the studies that assessed CMJ was 2,435, while for males it was only 874. This may suggest a diminished PJT efficiency in females calling into question their relative trainability in comparison to males. Alternatively, it is possible that the intensity of type of jumps prescribed were not the most appropriate. Regrettably, moderator analyses were precluded for certain dosing variables of PJT, due to the low number of studies per moderator category, including type of jump. If sex differences are related to the menstrual cycle, this is unclear at present, although the menstrual cycle would not affect athletic performance proxies such as sprint, jump, and force–velocity profile [174–176], especially with trained athletes, who are already used to coping with training under the menstrual cycle-related fluctuations.

Nonetheless, our meta-analysis moderated by the sex of the participants indicated non-significant differences between males and females. For the comparison between pre-PHV and post-PHV groups involved in PJT, no significant difference was found between the sexes. Further, for pre-PHV and post-PHV groups compared to controls, no significant differences were found between males and females. Moreover, results among all eight outcome measures included in the meta-analyses (Table 5) remained consistent for ES and p-values when a sex-based sensitivity analysis was performed. Overall, recommendations regarding the role of sex in adaptive responses to PJT in youth populations are rather limited. Indeed, in our meta-analysis, a comparison of results between studies that included males and females was conducted although only for CMJ, as the number of studies available for analysis for other outcomes was insufficient.

#### Programme Duration (Number of Weeks and Total Number of Training Sessions)

A comparison of results between studies with different programme durations was conducted, although this was only done for RSI and CMJ. We observed no significant effect for programme duration. In relation to RSI, pre-PHV and post-PHV provided data from seven and three groups in studies conducted over periods of ≤ 6 weeks and > 6 weeks respectively. Although the results were not significantly different (*p* = 0.106) between the ≤ 6 weeks (ES = 0.06) and > 6 weeks (ES = − 0.35) subgroups, four of seven groups involved in ≤ 6 weeks of PJT demonstrated a favourable effect in post-PHV. This resulted in a mean improvement of 4.9% over the pre-PHV participants. In contrast, all three groups involved in training for > 6 weeks showed a favourable effect in pre-PHV, with a mean improvement of 23.6% over the post-PHV participants. These results could suggest that over longer periods of PJT (i.e., > 6 weeks), pre-PHV participants may develop greater RSI improvements compared to their post-PHV counterparts. However, considering the potentially greater effects of power-strength training on physical fitness (i.e., jump, sprint) of untrained compared to trained youth [120], and since the participants’ experience with PJT before interventions was rarely well documented, it is difficult to determine if a greater RSI adaptive response to PJT may be moderated by previous exposure to this type of training (i.e., training history) compared to maturity. Relatedly, it is difficult to attribute these observations to optimal progressive overload during PJT. Firstly, this is because there is a lack of studies addressing optimization strategies for progressive overload in PJT [35, 36]. Secondly, amongst the studies that applied PJT for different durations, relatively similar progressive overload strategies were utilised through the manipulation of volume, technique, or a combination of both (Table 4). Accordingly, further studies are needed to elucidate optimal PJT progressions and to determine how these might interact with maturity to help youth athletes to avoid reaching stagnation following initial adaptation.

### Certainty of Evidence (GRADE)

For most outcomes, we would provide a *weak* recommendation in favour of PJT compared to controls (involving both sport-specific active controls and active controls involved in physical education classes), while for very low certainty cases, *no recommendation* would be advisable. For all comparisons not analysed meta-analytically, a very low certainty of evidence is suggested. Overall, low to very low certainty of evidence is currently apparent for PJT versus control studies and very low in relation to pre-PHV versus post-PHV participants exposed to PJT.

### Limitations and Recommendations for Future Research

Firstly, a relatively small number of available studies (*n* = 11) were included. Although a considerable number of PJT studies were found (Fig. 1), most of these were excluded at the eligibility stage because they did not report participants’ maturity status. Future studies are encouraged to report participants’ maturity status so that more accurate inferences on the adaptability of youths to exercise can be established. Secondly, a priori we considered maturity categories of ‘pre-PHV’ (i.e., < − 1.0 y PHV), ‘mid-PHV’ (i.e., − 1.0 to 1.0 y PHV), and ‘post-PHV’ (i.e., > 1.0 y PHV) [48, 51, 67] (for the full planned procedures, see the Registration section). However, a posteriori, there was an insufficient number of studies per outcome and maturity category, and this precluded the use of the ‘pre-PHV’, ‘mid-PHV’, and ‘post-PHV’ categories. Thirdly, moderator analyses were planned. However, only RSI and/or CMJ were available for such an evaluation. Further, some moderator analyses were precluded for certain dosing variables of PJT, due to the low number of studies per moderator category, including training frequency, type of jump, and studies that delivered PJT only or in conjunction with other training interventions. Fourthly, due to the limited number of studies, the analysis of the effects of PJT on outcomes such as body composition [29, 123, 124], cardiovascular fitness and health-related outcomes [177, 178] was precluded. Fifthly, the analysis for a potential *ceiling* effect was precluded, since most studies provided no report of participants’ previous experience with/exposure to PJT training (and training in general). Future studies should compare pre-PHV and post-PHV participants considering the amount of previous exposition to a certain intervention, including PJT.

### Implications of the Findings for Sport Practice

Evidence-based practical recommendations for PJT programming to improve the physical fitness of youths is provided in Table 7. However, an evidence-based proposal for optimal PJT programming according to maturity was precluded, due to the limitations of the currently available literature (previously exposed in this systematic review). Nonetheless, the information provided in Table 7 may provide a general framework for PJT programming that would allow safe and effective interventions for physical fitness improvements (compared to maturity-matched controls), irrespective of the maturity status of male and female participants.

**Table 7:** Evidence-based practical recommendations for PJT programming

	Freq	Dur	Int	BH	NTJ	Tply	Comb	RBS	RBR	RBTS ^a^	Tsurf	PO	Repl	Taper
Sport-specific performance (3)^b^	2	6–7	Max	20–60, optimal^c^	720–1,008 (120–168) ^d^	Mix, DJ	No	30–120	3–15	48–96	Grass, mix	V, no PO	Yes–No	Yes–No
Maximal dynamic strength (4)	1–2	6–36	Max	5–60	840–10,800 (120–300)	Mix, DJ	No	30–120	5–15	48–96	Grass, parquet, mix	T, V, no PO	Yes–No	Yes–No
Change of direction speed time (4)	1–2	6–36	Max, mix	5–60	840–10,800 (120–315)	Mix, DJ	No	5–90	5–15	48–96	Grass, mix	T, V, no PO	Yes–No	Yes–No
Linear sprinting speed time (6)	2	6–8	Max	5–60	600–1,590 (92–265)	Mix, DJ	No	30–120	5–15	48–96	Grass, parquet, mix	T, V, no PO	Yes–No	Yes–No
Horizontal jump distance (4)	1–2	6–36	Max	20–60, optimal	720–10,800 (120–300)	Mix, DJ	No	30–120	3–15	48–96	Grass, mix	T, V, no PO	Yes–No	Yes–No
Squat jump height (4)	2–3	6–8	Max, mix	5–60	958–3,510 (160–439)	Mix	No	30–120	5–15	48–96	Wood, parquet, mix	T, V, Int	Yes–No	Yes–No
Reactive strength index (7)	2	4–8	Max, mix	5–60, optimal	740–2,204 (120–315)	Mix, DJ	No	5–120	5–15	48–96	Grass, parquet, mix	T, V, no PO	Yes–No	Yes–No
Countermovement jump height (7)	2–3	6–8	Max, mix	5–60	600–3,510 (92–439)	Mix, DJ	No	5–120	5–15	48–96	Grass, wood, parquet, mix	T, V, Int, no PO	Yes–No	Yes–No

Abbreviations ordered alphabetically: *BH*: box or obstacle height (cm) used in PJT exercises (only applicable when drop jumps or similar jump drills are included); *Comb*: combined PJT exercises with other type of exercise (e.g., other than regular training practice for those who were athletes); *DJ*: drop jump; Duration: total duration (weeks) of PJT interventions; *Freq*: frequency of PJT training (sessions per week); *Int*: intensity of PJT intervention exercises; Max: maximal; NTJ: number of total jumps (e.g., foot contacts per leg). In some studies, jump repetitions were prescribed as distance or time. For this review, 1-m and 1-s were considered equivalent to 1 repetition; *PO*: progressive overload (either as intensity, type of exercise, volume, or a combination of these); *RBR*: rest between repetitions (seconds); *RBS*: rest between sets (seconds); *RBTS*: rest between PJT sessions (hours); *Repl*: replaced part of their regular activities (e.g., warm-up before a regular soccer training session) with PJT. If “No”, this implies that participants added PJT to their regular training schedule; T: technique or type of exercise; *Tply*: type of PJT exercises; *Tsurf*: type of surface during PJT sessions; *V*: volume (i.e., number) of jumps

^a^Applicable to training sessions within a given week

^b^Values in parenthesis represents the number of studies providing data for the outcome

^c^A box height allowing maximal reactive strength

^d^Values in parenthesis denote the number of jumps per week

An effective dose of PJT would involve ≥ 4 weeks of intervention, 1–2 sessions per week, incorporating multi-type or single-type [e.g., drop jump]) jump exercises. It is recommended to start with a reduced number of jumps per week (e.g., 92) and then progressively increase this number (e.g., by 10% per week). Of note, a total minimal dose of 600–1,000 jumps seems effective to improve most physical fitness outcomes. A progression-variation of jump type-techniques is also advised, particularly for long-term interventions. Regarding the intensity of PJT exercises, a sound technique of jump execution is advised to be attained before reaching high-maximal jump efforts. An inter-set rest of ≤ 120 s seems adequate, and shorter inter-set rest periods may also be effective and would allow reduction of the total duration of training sessions. For jump exercises requiring an inter-repetition rest-pause (e.g., drop jumps), 3–15 s seem adequate. For inter-session recovery, ≥ 48 h may be advised. Surfaces such as grass, wood, parquet, or a combination of surfaces, seem safe and effective to perform jump exercises. Current evidence suggests that PJT is effective either introduced as an additional load or to replace standard training (or if a taper approach is applied). However, practitioners are advised to take such a decision depending on the fitness characteristics of the participants and their current level of training and competition. It is advised that PJT be incorporated into a comprehensive multicomponent training approach for youths, with long-term training and performance development aims [75, 179, 180].

## Conclusion

Compared to control participants, pre- and post-PHV youths performing PJT experienced improved maximal dynamic strength, linear sprint speed, horizontal jump distance, RSI, and SSP (i.e., soccer ball kicking and dribbling velocity). These effects seem to occur independently of maturity status, as both pre-PHV and post-PHV participants achieved similar improvements following PJT interventions for most outcomes. However, several methodological issues (e.g., low sample sizes and the pooling of maturity categories) preclude the attainment of more robust recommendations at the current time. To address this issue in future studies with youth and youth athletes, the measurement of maturity status through skeletal age, Tanner stages, or anthropometric assessment methods is key and should be systematically reported in future studies with youth and youth athletes.

## Registration

The protocol for this systematic review with meta-analysis was published in the Open Science platform (OSF) on March 8, 2022. Link to project: https://osf.io/nd6w7/. Link to registration: https://osf.io/8dybe.

## Supplementary Information

**Additional file 1**. **Figures S1 to S24**, with meta-analyses for participants pre-PHV (PJT vs controls), post-PHV (PJT vs controls), and PJT (pre-PHV vs post-PHV).

**Additional file 2**. Characterization of activities performed by the control groups during the intervention period.

**Additional file 3**. Meta-analyses according to moderators: participants’ sex, PJT programme duration (number of weeks and total number of training sessions), total number of jumps, and studies methodological quality.

**Additional file 4. Table S1**. Search strategies (code line) for each database and background of search history.

**Additional file 5. Table S2**. Exclusion reasons for studies included in the preliminary qualitative synthesis.

## Data Availability

All data generated or analysed during this study are included in the article as Table(s), Figure(s), and/or Electronic Supplementary Material(s). Any other data requirement can be directed to the corresponding author upon reasonable request.

## Abbreviations

CI: Confidence interval
CMJ: Countermovement jump
COD: Change of direction
ES: Effect size
GRADE: Grading of recommendations assessment, development, and evaluation
OSF: Open Science platform
PEDro: Physiotherapy evidence database
PHV: Age at peak height velocity
PICOS: Participants, intervention, comparators, outcomes, and study design
PJT: Plyometric jump training
PRISMA: Preferred reporting items for systematic reviews and meta-analyses
RSI: Reactive strength index
SJ: Squat jump
SSC: Stretch–shortening cycle
SSP: Sport-specific performance
