# Supplementary material for: Plyometric-Jump Training Effects on Physical Fitness and Sport-Specific Performance According to Maturity: A Systematic Review with Meta-analysis
Source: Sports Med Open. 2023 Apr 10;9:23. doi: 10.1186/s40798-023-00568-6 (PMC10086091; doi:10.1186/s40798-023-00568-6)
Supplement: Supplementary file 1 — Additional file 1. Figures S1 to S24, with meta-analyses for participants pre-PHV (PJT vs controls), post-PHV (PJT vs controls), and PJT (pre-PHV vs post-PHV). [file 40798_2023_568_MOESM1_ESM.doc]

**Electronic Supplementary Material Figures S1 to S24**

**Article title**:

Plyometric-jump training effects on physical fitness and sport-specific performance according to maturity: A systematic review with meta-analysis

**Author names**:

Rodrigo Ramirez-Campillo, Andrew Sortwell, Jason Moran, José Afonso, Filipe Manuel Clemente, Rhodri S. Lloyd, Jon L. Oliver, Jason Pedley, Urs Granacher

**Affiliation and e-mail of the corresponding author**:

Prof. Urs Granacher, PhD

University of Freiburg

Department of Sport and Sport Science

Exercise and Human Movement Science

Sandfangweg 4

79102 Freiburg i. Br.

Germany

Email: urs.granacher@sport.uni-freiburg.de

**Abbreviations used in the file:**

PHV: age of peak height velocity; COD: change-of-direction; PJT: plyometric-jump training; 5RM: five repetition maximum.

**Meta-analyses for pre-PHV participants: PJT compared to controls**

Three studies provided data for sport-specific performance, involving six PJT pre-PHV groups (n=54) and six control pre-PHV groups (n=54). Results showed a significant small effect for the PJT groups compared to the control groups (ES = 0.55; 95% CI = 0.18 to 0.91; *p =* 0.004; Electronic Supplementary Material Fig. S1; *I*^2^ = 0.0%). Of note, studies involved sport-specific testing for ball kicking velocity/distance, except the study from Asadi et al. that involved dribbling velocity. However, when removed from the analysis, the results remained consistent (ES = 0.57; p = 0.006; I2 = 0.0%).

Electronic Supplementary Material Fig. S1. Forest plot for changes regarding sport-specific performance (e.g., ball kicking velocity) after plyometric jump training (PJT) compared to controls in participants at pre-PHV. Forest plot values are shown effect sizes (Hedges’ g) with 95% confidence intervals (CI). Black squares: individual studies. Its size represent their relative weights. White rhomboid: summary value. Note: letter at the end of a study (e.g., Martin et al., 2021 a) denotes that different experimental groups were included. PHV: age at peak height velocity.

Four studies provided data for maximal dynamic strength (e.g., 5RM back squat), involving four PJT pre-PHV groups (n=77) and four control pre-PHV groups (n=91). Results showed a significant small effect for the PJT groups compared to the control groups (ES = 0.35; 95% CI = 0.05 to 0.65; *p =* 0.022; Electronic Supplementary Material Fig. S2; *I*^2^ = 0.0%).

Electronic Supplementary Material Fig. S2. Forest plot for changes regarding maximal dynamic strength (e.g., 5RM back squat) after plyometric jump training (PJT) compared to controls in participants at pre-PHV. Forest plot values are shown effect sizes (Hedges’ g) with 95% confidence intervals (CI). Black squares: individual studies. Its size represent their relative weights. White rhomboid: summary value. PHV: age at peak height velocity.

Four studies provided data for COD speed time (e.g., Illinois test time), involving four pre-PHV (n=65) and four pre-PHV (n=67) PJT and control groups, respectively. Results showed a moderate non-significant (although near significance) effect for the PJT groups compared to the control groups (ES = 0.62; 95% CI = -0.06 to 1.31; *p =* 0.075; Electronic Supplementary Material Fig. S3; *I*^2^ = 71.2%).

Electronic Supplementary Material Fig. S3. Forest plot for changes regarding COD speed time (e.g., Illinois test) after plyometric jump training (PJT) compared to controls in participants at pre-PHV. Forest plot values are shown effect sizes (Hedges’ g) with 95% confidence intervals (CI). Black squares: individual studies. Its size represent their relative weights. White rhomboid: summary value. PHV: age at peak height velocity.

Six studies provided data for linear sprinting speed time (e.g., 20-m sprint), involving six pre-PHV (n=81) and six pre-PHV (n=90) PJT and control groups, respectively. Results showed a significant small effect for the PJT groups compared to the control groups (ES = 0.38; 95% CI = 0.09 to 0.68; *p =* 0.011; Figure S4; *I*^2^ = 0.0%).

Electronic Supplementary Material Fig. S4. Forest plot for changes regarding linear sprinting speed time (e.g., 10-m sprint) after plyometric jump training (PJT) compared to control in participants at pre-PHV. Forest plot values are shown effect sizes (Hedges’ g) with 95% confidence intervals (CI). Black squares: individual studies. Its size represent their relative weights. White rhomboid: summary value. PHV: age at peak height velocity.

Four studies provided data for horizontal jump distance (e.g., standing long jump test), involving seven pre-PHV (n=79) and seven pre-PHV (n=87) PJT and control groups, respectively. Results showed a significant small effect for the PJT groups compared to the control groups (ES = 0.42; 95% CI = 0.12 to 0.72; *p =* 0.006; Figure S5; *I*^2^ = 0.0%).

Electronic Supplementary Material Fig. S5. Forest plot for changes regarding horizontal jump distance (e.g., standing long jump test) after plyometric jump training (PJT) compared to controls in participants at pre-PHV. Forest plot values are shown effect sizes (Hedges’ g) with 95% confidence intervals (CI). Black squares: individual studies. Its size represent their relative weights. White rhomboid: summary value. Note: letter at the end of a study (e.g., Martin et al., 2021 a) denotes that different experimental groups were included. PHV: age at peak height velocity.

Four studies provided data for squat jump height, involving four pre-PHV controls (n=63) and PJT (n=56) groups. Results showed a significant small effect favouring the PJT groups (ES = 0.46; 95% CI = 0.11 to 0.82; *p =* 0.011; Figure S6; *I*^2^ = 0.0%).

Electronic Supplementary Material Fig. S6. Forest plot for changes regarding squat jump height after plyometric jump training (PJT) compared to controls in participants at pre-PHV. Forest plot values are shown effect sizes (Hedges’ g) with 95% confidence intervals (CI). Black squares: individual studies. Its size represent their relative weights. White rhomboid: summary value. PHV: age at peak height velocity.

Seven studies provided data for reactive strength index (e.g., mm.ms^-1^), involving ten pre-PHV control (n=126) and PJT (n=126) groups. Results showed a significant small effect favouring PJT (ES = 0.57; 95% CI = 0.33 to 0.82; *p <* 0.001; Figure S7; *I*^2^ = 0.0%).

Electronic Supplementary Material Fig. S7. Forest plot for changes regarding reactive strength index (e.g., mm.ms^-1^) after plyometric jump training (PJT) compared to controls in participants at pre-PHV. Forest plot values are shown effect sizes (Hedges’ g) with 95% confidence intervals (CI). Black squares: individual studies. Its size represent their relative weights. White rhomboid: summary value. Note: letter at the end of a study (e.g., Martin et al., 2021 a) denotes that different experimental groups were included. PHV: age at peak height velocity.

Seven studies provided data for countermovement jump height, involving seven pre-PHV control (n=100) and PJT (n=95) groups. Results showed a small non-significant (although near significance) effect for the PJT groups compared to the control groups (ES = 0.50; 95% CI = -0.08 to 1.08; *p =* 0.088; Figure S8; *I*^2^ = 73.4%).

Electronic Supplementary Material Fig. S8. Forest plot for changes regarding countermovement jump height after plyometric jump training (PJT) compared to controls in participants at pre-PHV. Forest plot values are shown effect sizes (Hedges’ g) with 95% confidence intervals (CI). Black squares: individual studies. Its size represent their relative weights. White rhomboid: summary value. PHV: age at peak height velocity.

**Meta-analyses for post-PHV participants: PJT compared to controls**

Three studies provided data for sport-specific performance, involving six post-PHV PJT (n=56) and six post-PHV control (n=56) groups. Results showed a significant moderate effect favouring PJT (ES = 0.82; 95% CI = 0.45 to 1.19; *p <* 0.001; Figure S9; *I*^2^ = 0.0%). Of note, studies involved sport-specific testing for ball kicking velocity/distance, except the study from Asadi et al. that involved dribbling velocity. However, when removed from the analysis, the results remained consistent (ES = 0.83; p < 0.001; I^2^ = 0.0%).

Electronic Supplementary Material Fig. S9. Forest plot for changes regarding sport-specific performance (e.g., ball kicking velocity) after plyometric jump training (PJT) compared to controls in participants at post-PHV. Forest plot values are shown effect sizes (Hedges’ g) with 95% confidence intervals (CI). Black squares: individual studies. Its size represent their relative weights. White rhomboid: summary value. Note: letter at the end of a study (e.g., Martin et al., 2021 a) denotes that different experimental groups were included. PHV: age at peak height velocity.

Four studies provided data for maximal dynamic strength (e.g., 5RM back squat), involving four PJT post-PHV (n=94) and four control post-PHV (n=79) groups. Results showed a significant small effect favouring PJT (ES = 0.46; 95% CI = 0.16 to 0.76; *p =* 0.003; Figure S10; *I*^2^ = 0.0%).

Electronic Supplementary Material Fig. S10. Forest plot for changes regarding maximal dynamic strength (e.g., 5RM back squat) after plyometric jump training (PJT) compared to controls in participants at post-PHV. Forest plot values are shown effect sizes (Hedges’ g) with 95% confidence intervals (CI). Black squares: individual studies. Its size represent their relative weights. White rhomboid: summary value. PHV: age at peak height velocity.

Four studies provided data for COD speed time (e.g., Illinois test time), involving four PJT post-PHV (n=81) and four control post-PHV (n=71) groups. Results showed a moderate non-significant (although near significance) effect for the PJT groups compared to the control groups (ES = 0.51; 95% CI = -0.18 to 1.21; *p =* 0.149; Figure S11; *I*^2^ = 74.8%).

Electronic Supplementary Material Fig. S11. Forest plot for changes regarding COD speed time (e.g., Illinois test) after plyometric jump training (PJT) compared to controls in participants at post-PHV. Forest plot values are shown effect sizes (Hedges’ g) with 95% confidence intervals (CI). Black squares: individual studies. Its size represent their relative weights. White rhomboid: summary value. PHV: age at peak height velocity.

Six studies provided data for linear sprinting velocity (e.g., 20-m sprint), involving six PJT post-PHV (n=83) and six control post-PHV (n=79) groups. Results showed a significant small effect favouring PJT (ES = 0.50; 95% CI = 0.19 to 0.81; *p =* 0.002; Figure S12; *I*^2^ = 1.9%).

Electronic Supplementary Material Fig. S12. Forest plot for changes regarding linear sprinting velocity (e.g., 10-m sprint) after plyometric jump training (PJT) compared to control in participants at post-PHV. Forest plot values are shown effect sizes (Hedges’ g) with 95% confidence intervals (CI). Black squares: individual studies. Its size represent their relative weights. White rhomboid: summary value. PHV: age at peak height velocity.

Four studies provided data for horizontal jump distance (e.g., standing long jump test), involving seven PJT post-PHV (n=95) and seven post-PHV (n=85) groups. Results showed a significant small effect favouring PJT (ES = 0.56; 95% CI = 0.27 to 0.84; *p <* 0.001; Figure S13; *I*^2^ = 0.0%).

Electronic Supplementary Material Fig. S13. Forest plot for changes regarding horizontal jump distance (e.g., standing long jump test) after plyometric jump training (PJT) compared to controls in participants at post-PHV. Forest plot values are shown effect sizes (Hedges’ g) with 95% confidence intervals (CI). Black squares: individual studies. Its size represent their relative weights. White rhomboid: summary value. Note: letter at the end of a study (e.g., Martin et al., 2021 a) denotes that different experimental groups were included. PHV: age at peak height velocity.

Four studies provided data for squat jump height, involving four post-PHV control (n=57) and four post-PHV PJT (n=53) groups. Results showed no effect between control and PJT groups (ES = 0.20; 95% CI = -0.17 to 0.57; *p =* 0.291; Figure S14; *I*^2^ = 0.0%).

Electronic Supplementary Material Fig. S14. Forest plot for changes regarding squat jump height after plyometric jump training (PJT) compared to controls in participants at post-PHV. Forest plot values are shown effect sizes (Hedges’ g) with 95% confidence intervals (CI). Black squares: individual studies. Its size represent their relative weights. White rhomboid: summary value. PHV: age at peak height velocity.

Seven studies provided data for reactive strength index (e.g., mm.ms^-1^), involving ten post-PHV control (n=118) and ten post-PHV PJT (n=120) groups. Results showed a significant small effect favouring PJT (ES = 0.40; 95% CI = 0.15 to 0.65; *p =* 0.002; Figure S15; *I*^2^ = 0.0%).

Electronic Supplementary Material Fig. S15. Forest plot for changes regarding reactive strength index (e.g., mm.ms^-1^) after plyometric jump training (PJT) compared to controls in participants at post-PHV. Forest plot values are shown effect sizes (Hedges’ g) with 95% confidence intervals (CI). Black squares: individual studies. Its size represent their relative weights. White rhomboid: summary value. Note: letter at the end of a study (e.g., Martin et al., 2021 a) denotes that different experimental groups were included. PHV: age at peak height velocity.

Seven studies provided data for countermovement jump height, involving seven post-PHV control (n=98) seven post-PHV PJT (n=94) groups. Results showed a small non-significant (although near significance) effect for the PJT groups compared to the control groups (ES = 0.36; 95% CI = -0.02 to 0.73; *p =* 0.061; Figure S16; *I*^2^ = 39.5%).

Electronic Supplementary Material Fig. S16. Forest plot for changes regarding countermovement jump height after plyometric jump training (PJT) compared to controls in participants at post-PHV. Forest plot values are shown effect sizes (Hedges’ g) with 95% confidence intervals (CI). Black squares: individual studies. Its size represent their relative weights. White rhomboid: summary value. PHV: age at peak height velocity.

**Meta-analyses for PJT participants: pre-PHV compared to post-PHV**

Three studies provided data for sport-specific performance, involving six pre-PHV (n=54) and six post-PHV (n=56) experimental groups. Results showed no effect for the PJT pre-PHV groups compared to the PJT post-PHV groups (ES = 0.11; 95% CI = -0.25 to 0.47; *p =* 0.565; Figure S17; *I*^2^ = 0.0%). %). Of note, studies involved sport-specific testing for ball kicking velocity/distance, except the study from Asadi et al. that involved dribbling velocity. However, when removed from the analysis, the results remained consistent (ES = 0.10; p = 0.627; I^2^ = 0.0%).

Electronic Supplementary Material Fig. S17. Forest plot for changes regarding sport-specific performance (e.g., ball kicking velocity) after plyometric jump training in participants at pre-PHV compared to post-PHV. Forest plot values are shown effect sizes (Hedges’ g) with 95% confidence intervals (CI). Black squares: individual studies. Its size represent their relative weights. White rhomboid: summary value. Note: letter at the end of a study (e.g., Martin et al., 2021 a) denotes that different experimental groups were included. PHV: age at peak height velocity.

Four studies provided data for maximal dynamic strength (e.g., 5RM back squat), involving four pre-PHV (n=77) and four post-PHV (n=94) experimental groups. Results showed no effect for the PJT pre-PHV groups compared to the PJT post-PHV groups (ES = -0.09; 95% CI = -0.39 to 0.21; *p =* 0.546; Figure S18; *I*^2^ = 0.0%).

Electronic Supplementary Material Fig. S18. Forest plot for changes regarding maximal dynamic strength (e.g., 5RM back squat) after plyometric jump training in participants at pre-PHV compared to post-PHV. Forest plot values are shown effect sizes (Hedges’ g) with 95% confidence intervals (CI). Black squares: individual studies. Its size represent their relative weights. White rhomboid: summary value. PHV: age at peak height velocity.

Four studies provided data for COD speed time (e.g., Illinois test time), involving four pre-PHV (n=65) and four post-PHV (n=81) experimental groups. Results showed a small significant effect for the PJT pre-PHV groups compared to the PJT post-PHV groups (ES = -0.42; 95% CI = -0.75 to -0.09; *p =* 0.012; Figure S19; *I*^2^ = 0.0%).

Electronic Supplementary Material Fig. S19. Forest plot for changes regarding COD speed time (e.g., Illinois test) after plyometric jump training in participants at pre-PHV compared to post-PHV. Forest plot values are shown effect sizes (Hedges’ g) with 95% confidence intervals (CI). Black squares: individual studies. Its size represent their relative weights. White rhomboid: summary value. PHV: age at peak height velocity.

Six studies provided data for linear sprinting speed time (e.g., 20-m sprint), involving six pre-PHV (n=81) and six post-PHV (n=83) experimental groups. Results showed no effect for the PJT pre-PHV groups compared to the PJT post-PHV groups (ES = 0.00; 95% CI = -0.30 to 0.30; *p =* 0.980; Figure S20; *I*^2^ = 0.0%).

Electronic Supplementary Material Fig. S20. Forest plot for changes regarding linear sprinting speed time (e.g., 10-m sprint) after plyometric jump training in participants at pre-PHV compared to post-PHV. Forest plot values are shown effect sizes (Hedges’ g) with 95% confidence intervals (CI). Black squares: individual studies. Its size represent their relative weights. White rhomboid: summary value. PHV: age at peak height velocity.

Four studies provided data for horizontal jump distance (e.g., standing long jump test), involving seven pre-PHV (n=79) and seven post-PHV (n=95) experimental groups. Results showed no effect for the PJT pre-PHV groups compared to the PJT post-PHV groups (ES = 0.08; 95% CI = -0.21 to 0.37; *p =* 0.583; Figure S21; *I*^2^ = 0.0%).

Electronic Supplementary Material Fig. S21. Forest plot for changes regarding horizontal jump distance (e.g., standing long jump test) after plyometric jump training in participants at pre-PHV compared to post-PHV. Forest plot values are shown effect sizes (Hedges’ g) with 95% confidence intervals (CI). Black squares: individual studies. Its size represent their relative weights. White rhomboid: summary value. Note: letter at the end of a study (e.g., Martin et al., 2021 a) denotes that different experimental groups were included. PHV: age at peak height velocity.

Four studies provided data for squat jump height, involving four pre-PHV (n=56) and four post-PHV (n=53) experimental groups. Results showed no effect for the PJT pre-PHV groups compared to the PJT post-PHV groups (ES = -0.13; 95% CI = -0.55 to 0.30; *p =* 0.558; Figure S22; *I*^2^ = 19.8%).

Electronic Supplementary Material Fig. S22. Forest plot for changes regarding squat jump height after plyometric jump training in participants at pre-PHV compared to post-PHV. Forest plot values are shown effect sizes (Hedges’ g) with 95% confidence intervals (CI). Black squares: individual studies. Its size represent their relative weights. White rhomboid: summary value. PHV: age at peak height velocity.

Seven studies provided data for reactive strength index (e.g., mm.ms^-1^), involving ten pre-PHV (n=126) and ten post-PHV (n=120) experimental groups. Results showed no effect for the PJT pre-PHV groups compared to the PJT post-PHV groups (ES = -0.12; 95% CI = -0.37 to 0.12; *p =* 0.330; Figure S23; *I*^2^ = 0.0%).

Electronic Supplementary Material Fig. S23. Forest plot for changes regarding reactive strength index (e.g., mm.ms^-1^) after plyometric jump training in participants at pre-PHV compared to post-PHV. Forest plot values are shown effect sizes (Hedges’ g) with 95% confidence intervals (CI). Black squares: individual studies. Its size represent their relative weights. White rhomboid: summary value. Note: letter at the end of a study (e.g., Martin et al., 2021 a) denotes that different experimental groups were included. PHV: age at peak height velocity.

Seven studies provided data for countermovement jump height, involving seven pre-PHV (n=95) and seven post-PHV (n=94) experimental groups. Results showed no effect for the PJT pre-PHV groups compared to the PJT post-PHV groups (ES = 0.10; 95% CI = -0.18 to 0.38; *p =* 0.499; Electronic Supplementary Material Fig. S24; *I*^2^ = 0.0%).

Electronic Supplementary Material Fig. S24. Forest plot for changes regarding countermovement jump height after plyometric jump training in participants at pre-PHV compared to post-PHV. Forest plot values are shown effect sizes (Hedges’ g) with 95% confidence intervals (CI). Black squares: individual studies. Its size represent their relative weights. White rhomboid: summary value. PHV: age at peak height velocity.
