# Supplementary material for: Plyometric-Jump Training Effects on Physical Fitness and Sport-Specific Performance According to Maturity: A Systematic Review with Meta-analysis
Source: Sports Med Open. 2023 Apr 10;9:23. doi: 10.1186/s40798-023-00568-6 (PMC10086091; doi:10.1186/s40798-023-00568-6)
Supplement: Supplementary file 2 — Additional file 2. Characterization of activities performed by the control groups during the intervention period. [file 40798_2023_568_MOESM2_ESM.docx]

**Electronic Supplementary Material File S1**

**Article title**:

Plyometric-jump training effects on physical fitness and sport-specific performance according to maturity: A systematic review with meta-analysis

**Author names**:

Rodrigo Ramirez-Campillo, Andrew Sortwell, Jason Moran, José Afonso, Filipe Manuel Clemente, Rhodri S. Lloyd, Jon L. Oliver, Jason Pedley, Urs Granacher

**Affiliation and e-mail of the corresponding author**:

Prof. Urs Granacher, PhD

University of Freiburg

Department of Sport and Sport Science

Exercise and Human Movement Science

Sandfangweg 4

79102 Freiburg i. Br.

Germany

Email: urs.granacher@sport.uni-freiburg.de

**File S1: Characterization of activities performed by the control groups during the intervention period**

| Asadi et al. 2018 [1] recruited sport-specific active controls. All athletes performed soccer specific training on three non-consecutive days (Monday, Wednesday, Friday), while the training groups performed plyometric training on the days in which soccer training was not completed (Tuesday, Saturday). |
| --- |
|  |
| Davies et al. 2021 [2] recruited active-controls. The control group consisted of students who would normally also be undertaking the same physical education class as the intervention group. |
|  |
| Heinonen et al. 2000 [3] recruited active-controls involved in school physical education classes. There were no differences between training and control schools as regards the curricular physical activity program. Controls and experimental participants had similar minutes of physical activity per day. |
|  |
| Lloyd et al. 2016 [4] recruited active-controls, involved in school physical education. The training groups completed two training sessions per week for 6 weeks instead of their regular physical education classes. Conversely, the control group continued with their physical education curricula. |
|  |
| Lloyd et al. 2012 [5] recruited active-controls involved in school physical education. The participants were divided into either an experimental or control within their respective age groups, based on their weekly school timetable for physical education lessons. |
|  |
| Moran et al. 2017 [6] recruited sport-specific active controls. The controls concurrently executed low intensity hockey skills work. |
|  |
| Ramirez-Campillo et al. 2019 [7] recruited sport-specific active controls. During the control phases, all participants maintained their regular soccer drills, while during experimental phases the players replaced ~11% of their technical-tactical drill sessions by plyometric training. |
| Romero et al. 2021 [8] recruited active-controls involved in school physical education classes that involved some sport-related activities, such as volleyball, soccer, or similar indoor-sports. The participants assigned to the experimental condition performed jump drills, replacing part of their physical education classes, twice per week. Participants in the control groups followed their regular physical education classes, twice per week. |
|  |
| Uzelac-Sciran et al. 2020 [9] recruited active-controls involved in school physical education. The participants were assigned to either a jump training program group (two jump training sessions per week for 8 weeks), or to a control group (continuing with regular, games-based physical education classes). |
|  |
| Vera-Asaoka et al. 2020 [10] recruited sport-specific active controls. The intervention groups replaced some technical drills with plyometric jump drills within the usual 90-min practice twice per week (Tuesday and Thursday) for 7 weeks, while the controls keep their regular soccer training schedule. |
|  |
| Vilela et al. 2021 [11] recruited sport-specific active controls, adding plyometric jump training drills to their regular volleyball training routine. The controls keep their normal volleyball training schedule of three weekly sessions. |
|  |

**REFERENCES**

1. Asadi A, Ramirez-Campillo R, Arazi H, Saez de Villarreal E. The effects of maturation on jumping ability and sprint adaptations to plyometric training in youth soccer players. J Sports Sci. 2018 Nov;36(21):2405-11.

2. Davies MJ, Drury B, Ramirez-Campillo R, Chaabane H, Moran J. Effect of plyometric training and biological maturation on jump and change of direction ability in female youth. J Strength Cond Res. 2021;35(10):2690-7.

3. Heinonen A, Sievänen H, Kannus P, Oja P, Pasanen M, Vuori I. High-impact exercise and bones of growing girls: a 9-month controlled trial. Osteoporosis international : a journal established as result of cooperation between the European Foundation for Osteoporosis and the National Osteoporosis Foundation of the USA. 2000;11(12):1010-7.

4. Lloyd RS, Radnor JM, De Ste Croix MBA, Cronin JB, Oliver JL. Changes in sprint and jump performances after traditional, plyometric, and combined resistance training in male youth pre- and post-peak height velocity. J Strength Cond Res. 2016;30(5):1239-47.

5. Lloyd RS, Oliver JL, Hughes MG, Williams CA. The effects of 4-weeks of plyometric training on reactive strength index and leg stiffness in male youths. J Strength Cond Res. 2012;26(10):2812-9.

6. Moran J, Sandercock GRH, Ramírez-Campillo R, Todd O, Collison J, Parry DA. Maturation-related effect of low-dose plyometric training on performance in youth hockey players. Pediatr Exerc Sci. 2017;29(2):194-202.

7. Ramirez-Campillo R, Alvarez C, Sanchez-Sanchez J, Slimani M, Gentil P, Chelly MS, et al. Effects of plyometric jump training on the physical fitness of young male soccer players: Modulation of response by inter-set recovery interval and maturation status. J Sport Sci. 2019:1-8.

8. Romero C, Ramirez-Campillo R, Alvarez C, Moran J, Slimani M, Gonzalez J, et al. Effects of maturation on physical fitness adaptations to plyometric jump training in youth females. J Strength Cond Res. 2021;35(10):2870-7.

9. Uzelac-Sciran T, Sarabon N, Mikulic P. Effects of 8-week jump training program on sprint and jump performance and leg strength in pre- and post-peak height velocity aged boys. J Sports Sci Med. 2020;19(3):547-55.

10. Vera-Assaoka T, Ramirez-Campillo R, Alvarez C, Garcia-Pinillos F, Moran J, Gentil P, et al. Effects of maturation on physical fitness adaptations to plyometric drop jump training in male youth soccer players. J Strength Cond Res. 2020;34(10):2760-8.

11. Vilela G, Caniuqueo-Vargas A, Ramirez-Campillo R, Hernandez-Mosqueira C, da Silva SF. Effects of plyometric training on explosive strength in pubescent girls volleyball players. Retos. 2021(40):41-6.
