# Supplementary material for: Plyometric-Jump Training Effects on Physical Fitness and Sport-Specific Performance According to Maturity: A Systematic Review with Meta-analysis
Source: Sports Med Open. 2023 Apr 10;9:23. doi: 10.1186/s40798-023-00568-6 (PMC10086091; doi:10.1186/s40798-023-00568-6)
Supplement: Supplementary file 3 — Additional file 3. Meta-analyses according to moderators: participants’ sex, PJT programme duration (number of weeks and total number of training sessions), total number of jumps, and studies methodological quality. [file 40798_2023_568_MOESM3_ESM.docx]

**Electronic Supplementary Material File S2**

**Article title**:

Plyometric-jump training effects on physical fitness and sport-specific performance according to maturity: A systematic review with meta-analysis

**Author names**:

Rodrigo Ramirez-Campillo, Andrew Sortwell, Jason Moran, José Afonso, Filipe Manuel Clemente, Rhodri S. Lloyd, Jon L. Oliver, Jason Pedley, Urs Granacher

**Affiliation and e-mail of the corresponding author**:

Prof. Urs Granacher, PhD

University of Freiburg

Department of Sport and Sport Science

Exercise and Human Movement Science

Sandfangweg 4

79102 Freiburg i. Br.

Germany

Email: urs.granacher@sport.uni-freiburg.de

**File S2: Meta-analyses according to moderators: participants’ sex, PJT programme duration (number of weeks and total number of training sessions), total number of jumps, and studies methodological quality**

**Participant’s sex:** Comparison of results between studies that included males and females were conducted, although only for CMJ, as the number of studies available for analysis for other outcomes was insufficient. For the comparison between PJT groups with pre-PHV and post-PHV, four and three groups provided data from studies conducted in male and female participants, respectively. Results were no significant different (p=0.552) between male (ES = 0.03; 95% CI = -0.32 to 0.37; I^2^ = 0.0%) and females (ES = 0.27; 95% CI = -0.44 to 0.98; I^2^ = 53.5%). For the comparison between pre-PHV participants involved in PJT compared to controls, four and three groups provided data from studies conducted in male and female participants, respectively. Results were no significant different (p=0.178) between male (ES = 0.87; 95% CI = -0.19 to 1.86; I^2^ = 84.9%) and females (ES = 0.12; 95% CI = -0.37 to 0.60; I^2^ = 0.0%). For the comparison between post-PHV participants involved in PJT compared to controls, four and three groups provided data from studies conducted in male and female participants, respectively. Results were no significant different (p=0.369) between male (ES = 0.51; 95% CI = -0.08 to 1.09; I^2^ = 59.9%) and females (ES = 0.16; 95% CI = -0.31 to 0.63; I^2^ = 0.0%).

**Programme duration (number of weeks and total number of training sessions):** Comparison of results between studies that included <7 weeks (12-13 total PJT sessions) and ≥7 weeks (14-24 total PJT sessions) were conducted, although only for RSI and CMJ, as the number of studies available for analysis for other outcomes was insufficient.

Regarding RSI, for the comparison between PJT groups with pre-PHV and post-PHV, seven and three groups provided data from studies conducted during ≤6 weeks and >6 weeks, respectively. Results were no significant different (p=0.106) between ≤6 weeks (ES = 0.06; 95% CI = -0.27 to 0.38; I^2^ = 0.0%) and >6 weeks (ES = -0.35; 95% CI = -0.72 to 0.02; I^2^ = 0.0%). For the comparison between pre-PHV participants involved in PJT compared to controls, seven and three groups provided data from studies conducted during ≤6 weeks and >6 weeks, respectively. Results were no significant different (p=0.063) between ≤6 weeks (ES = 0.36; 95% CI = 0.04 to 0.69; I^2^ = 0.0%) and >6 weeks (ES = 0.86; 95% CI = 0.45 to 1.26; I^2^ = 9.5%). For the comparison between post-PHV participants involved in PJT compared to controls, seven and three groups provided data from studies conducted during ≤6 weeks and >6 weeks, respectively. Results were no significant different (p=0.949) between ≤6 weeks (ES = 0.38; 95% CI = 0.05 to 0.72; I^2^ = 0.0%) and >6 weeks (ES = 0.36; 95% CI = -0.33 to 1.05; I^2^ = 68.5%).

Regarding CMJ, for the comparison between PJT groups with pre-PHV and post-PHV, three and four groups provided data from studies conducted during <7 weeks and ≥7 weeks, respectively. Results were no significant different (p=0.520) between <7 weeks (ES = 0.26; 95% CI = -0.24 to 0.77; I^2^ = 0.0%) and ≥7 weeks (ES = 0.05; 95% CI = -0.39 to 0.48; I^2^ = 36.5%). For the comparison between pre-PHV participants involved in PJT compared to controls, three and four groups provided data from studies conducted during <7 weeks and ≥7 weeks, respectively. Results were no significant different (p=0.214) between <7 weeks (ES = 1.26; 95% CI = -0.39 to 2.92; I^2^ = 88.1%) and ≥7 weeks (ES = 0.19; 95% CI = -0.14 to 0.53; I^2^ = 0.0%). For the comparison between post-PHV participants involved in PJT compared to controls, three and four groups provided data from studies conducted during <7 weeks and ≥7 weeks, respectively. Results were no significant different (p=0.184) between <7 weeks (ES = 0.80; 95% CI = -0.09 to 1.69; I^2^ = 63.1%) and ≥7 weeks (ES = 0.16; 95% CI = -0.17 to 0.49; I^2^ = 0.0%).

**Programme total number of jumps:** Only RSI and CMJ outcomes were available for the moderator analysis according to programme total number of jumps. The number of studies available for analysis for other outcomes was insufficient. Of note, for CMJ, the results derived from moderator analyses according to programme total number of jumps were the same as the results derived from moderator analyses according to participant’s sex, with males and females performing <1,336 total jumps and ≥1,336 total jumps, respectively.

Comparison of results between studies that included <1,000 jumps and ≥1,000 jumps (i.e., median of total jumps among studies that provided data for RSI). For the comparison between PJT groups with pre-PHV and post-PHV, seven and three groups provided data from studies using <1,000 jumps and ≥1,000 jumps, respectively. Results were no significant different (p=0.272) between <1,000 jumps (ES = 0.01; 95% CI = -0.30 to 0.32; I^2^ = 0.0%) and ≥1,000 jumps (ES = -0.32; 95% CI = -0.81 to 0.18; I^2^ = 30.6%). For the comparison between pre-PHV participants involved in PJT compared to controls, seven and three groups provided data from studies using <1,000 jumps and ≥1,000 jumps, respectively. Results were no significant different (p=0.987) between <1,000 jumps (ES = 0.53; 95% CI = 0.22 to 0.84; I^2^ = 0.0%) and ≥1,000 jumps (ES = 0.52; 95% CI = -0.13 to 1.18; I^2^ = 58.5%). For the comparison between post-PHV participants involved in PJT compared to controls, seven and three groups provided data from studies using <1,000 jumps and ≥1,000 jumps, respectively. Results were no significant different (p=0.319) between <1,000 jumps (ES = 0.50; 95% CI = 0.19 to 0.81; I^2^ = 0.0%) and ≥1,000 jumps (ES = 0.21; 95% CI = -0.28 to 0.69; I^2^ = 19.9%).

**Studies methodological quality:** Comparison of results between studies with 5 points and 6 points in the PEDro scale were conducted, although only for RSI, as the number of studies available for analysis for other outcomes was insufficient. For the comparison between PJT groups with pre-PHV and post-PHV, four and six groups provided data from studies with moderate and high methodological quality, respectively. Results were no significant different (p=0.069) between moderate (ES = -0.33; 95% CI = -0.66 to 0.00; I^2^ = 0.0%) and high-quality (ES = 0.13; 95% CI = -0.24 to 0.48; I^2^ = 0.0%). For the comparison between pre-PHV participants involved in PJT compared to controls, four and six groups provided data from studies with moderate and high methodological quality, respectively. Results were no significant different (p=0.185) between moderate (ES = 0.72; 95% CI = 0.39 to 1.06; I^2^ = 0.0%) and high-quality (ES = 0.39; 95% CI = 0.02 to 0.76; I^2^ = 0.0%). For the comparison between post-PHV participants involved in PJT compared to controls, four and six groups provided data from studies with moderate and high methodological quality, respectively. Results were no significant different (p=0.234) between moderate (ES = 0.26; 95% CI = -0.08 to 0.60; I^2^ = 0.0%) and high-quality (ES = 0.56; 95% CI = 0.20 to 0.93; I^2^ = 0.0%).
