# Supplementary material for: Plyometric-Jump Training Effects on Physical Fitness and Sport-Specific Performance According to Maturity: A Systematic Review with Meta-analysis
Source: Sports Med Open. 2023 Apr 10;9:23. doi: 10.1186/s40798-023-00568-6 (PMC10086091; doi:10.1186/s40798-023-00568-6)
Supplement: Supplementary file 4 — Additional file 4. Table S1. Search strategies (code line) for each database and background of search history. [file 40798_2023_568_MOESM4_ESM.docx]

**Electronic Supplementary Material Table S1**

**Article title**:

Plyometric-jump training effects on physical fitness and sport-specific performance according to maturity: A systematic review with meta-analysis

**Author names**:

Rodrigo Ramirez-Campillo, Andrew Sortwell, Jason Moran, José Afonso, Filipe Manuel Clemente, Rhodri S. Lloyd, Jon L. Oliver, Jason Pedley, Urs Granacher

**Affiliation and e-mail of the corresponding author**:

Prof. Urs Granacher, PhD

University of Freiburg

Department of Sport and Sport Science

Exercise and Human Movement Science

Sandfangweg 4

79102 Freiburg i. Br.

Germany

Email: urs.granacher@sport.uni-freiburg.de

Table S1. Search strategies (code line) for each database and background of search history.

| **Date of the search** | April, 2017 | May, 2019 | August, 2021 |
| --- | --- | --- | --- |
| **Databases** | PubMed | PubMed, WOS (Core Collection), Scopus | PubMed, WOS (Core Collection) ^a^, Scopus |
| **Keywords** | “plyometric”, “training” | “ballistic”, “complex”, “cycle”, “explosive”, “force”, “plyometric”, “shortening”, “stretch”, “training”, “velocity” | “ballistic”, “complex”, “cycle”, “explosive”, “force”, “jump”, “plyometric”, “power”, “shortening”, “stretch”, “training”, “velocity” |
| **Database fields for the search** | All | PubMed: all  WOS: all  Scopus: title, abstract, keywords | PubMed: all ^b^  WOS: all ^b^  Scopus: title, abstract, keywords ^b^ |
| **Restrictions for the search** | None | None | None |
| **Examples of search strategy code line** | PubMed: "plyometric exercise"[MeSH Terms] OR ("plyometric"[All Fields] AND "exercise"[All Fields]) OR "plyometric exercise"[All Fields] OR ("plyometric"[All Fields] AND "training"[All Fields]) OR "plyometric training"[All Fields]  WOS: (ALL=(plyometric)) AND ALL=(training)  Scopus: TITLE-ABS-KEY ( plyometric AND training ) | | |
| ^a^: except for the keywords “jump” and “power” searched in all Web of Science (WOS) databases.  ^b^: except for the keywords “jump” and “power” searched in the database field TITLE (a very poor efficiency was obtained in the search for results with the incorporation of other database fields). | | | |
