# Supplementary material for: Plyometric-Jump Training Effects on Physical Fitness and Sport-Specific Performance According to Maturity: A Systematic Review with Meta-analysis
Source: Sports Med Open. 2023 Apr 10;9:23. doi: 10.1186/s40798-023-00568-6 (PMC10086091; doi:10.1186/s40798-023-00568-6)
Supplement: Supplementary file 5 — Additional file 5. Table S2. Exclusion reasons for studies included in the preliminary qualitative synthesis. [file 40798_2023_568_MOESM5_ESM.docx]

**Electronic Supplementary Material Table S2**

**Article title**:

Plyometric-jump training effects on physical fitness and sport-specific performance according to maturity: A systematic review with meta-analysis

**Author names**:

Rodrigo Ramirez-Campillo, Andrew Sortwell, Jason Moran, José Afonso, Filipe Manuel Clemente, Rhodri S. Lloyd, Jon L. Oliver, Jason Pedley, Urs Granacher

**Affiliation and e-mail of the corresponding author**:

Prof. Urs Granacher, PhD

University of Freiburg

Department of Sport and Sport Science

Exercise and Human Movement Science

Sandfangweg 4

79102 Freiburg i. Br.

Germany

Email: urs.granacher@sport.uni-freiburg.de

| Table S2. Exclusion reasons for studies included in the preliminary qualitative synthesis. | |
| --- | --- |
| **Article** | **Reason** |
| Bassey et al. 1998 [1] | Participants/comparison-related, i.e. compared age groups >18 years of age. |
| Ciacci and Bartolomei 2018 [2] | Participants/comparison-related, i.e., compared different age groups (under 17 and under 19 years of competitive age categories for soccer), although they did not report maturity. |
| Dallas et al. 2020 [3] | Participants/comparison-related, i.e., compared different age groups (<14 vs <9), although they did not report maturity. Additionally, age groups practiced other sports (taekwondo vs gymnastics, respectively). |
| Falces-Prieto et al. 2021[4] | Participants/comparison-related, i.e., compared different age groups (under 16 and under 19 years competitive age categories for soccer), although they did not report maturity. |
| Foss et al. 2018 [5] | Outcome-related, i.e., did not report physical fitness and/or sport-specific performance outcomes (i.e., focus on injuries). |
| Franchi et al. 2019 [6] | Participants/comparison-related, i.e. compared age groups >18 years of age. |
| Ha et al. 2014 [7] | Participants/comparison-related, i.e. compared age groups <18 vs. >18 years of age (i.e., data was collected in youth participating in the intervention groups, and in adults, but the adults were not involved in the intervention), and no information was provided regarding maturity status. |
| Hunnicutt et al. 2016 [8] | Participants/comparison-related, i.e. compared age groups >18 years of age. |
| Keiner et al. 2014 [9] | Participants/comparison-related, i.e., compared different age groups, although from the same maturity category (i.e., pre-PHV). |
| Marina and Jemni 2014 [10] | Participants/comparison-related, i.e., compared one single group of participants over two training/competition seasons, and although maturity changed across time, most participants remained pre-menarchial. |
| Marta et al. 2014 [11] | Participants/comparison-related, i.e. compared Tanner I and Tanner II (as per our protocol, both correspond to the same maturity category; full explanation available in the main manuscript, section 2.6.1). Although the authors reported that “…it seems that the variable sex had no influence on training-induced strength…or endurance…”, a considerable difference was noted in the distribution of boys and girls between maturation groups, i.e. 41 participants for the plyometric-jump training intervention, involving 25 participants in Tanner I (14 boys; 11 girls) and 16 in Tanner II (5 boys; 11 girls). |
| Michel et al. 2014 [12] | Participants/comparison-related, i.e., compared different age groups, although from the same maturity category (i.e., pre-menarchial). |
| Mero et al. 2021 [13] | Intervention-related, i.e., training interventions, were different between age/maturity groups. |
| Pardos-Mainer 2017 [14] | Participants/comparison-related, i.e. compared age groups with different sex (i.e., male = 16.3 y, post-PHV; female = 12.6 y, pre-PHV). |
| Peña-Gonzalez 2019 [15] | Intervention-related, i.e., exclusion criteria of intervention, mixing RT and PJT exercises, with <50% total training exercises from jump drills. |
| Radnor et al. 2017 [16] | Outcome-related, i.e., the authors performed a secondary statistical analysis from previously published data. |
| Saez de Villarreal 2010 [17] | Participants/comparison-related, i.e. compared age groups >18 years of age. |
| Saez de Villarreal 2010 [18] | Participants/comparison-related, i.e. compared age groups >18 years of age. |
| Vassil and Bazanovk 2012 [19] | Participants/comparison-related, i.e. compared age groups with different sex and similar maturity (i.e., male = 17.0 y, post-PHV; female = 14.4 y, post-PHV). |
| Verma et al. 2015 [20] | Participants/comparison-related, i.e. authors reported groups of different maturity status, but without a well-defined or standardized method, i.e. authors indicated “pre-pubertal” and “pubertal” categories, although without further description of maturity assessment methods. |
| PHV: age of peak height velocity. | |

**REFERENCES**

1. Bassey EJ, Rothwell MC, Littlewood JJ, Pye DW. Pre- and postmenopausal women have different bone mineral density responses to the same high-impact exercise. J Bone Miner Res. 1998;13(12):1805-13.

2. Ciacci S, Bartolomei S. The effects of two different explosive strength training programs on vertical jump performance in basketball. J Sports Med Phys Fitness. 2018 Oct;58(10):1375-82.

3. Dallas GC, Pappas P, Ntallas CG, Paradisis GP, Exell TA. The effect of four weeks of plyometric training on reactive strength index and leg stiffness is sport dependent. J Sports Med Phys Fitness. 2020;60(7):979-84.

4. Falces-Prieto M, Raya-González J, de Villarreal ES, Rodicio-Palma J, Iglesias-García FJ, Fernández FTG. Effects of combined plyometric and sled training on vertical jump and linear speed performance in young soccer players. Retos. 2021;42:228-35.

5. Foss KDB, Thomas S, Khoury JC, Myer GD, Hewett TE. A school-based neuromuscular training program and sport-related injury incidence: a prospective randomized controlled clinical trial. J Athl Train. 2018;53(1):20-8.

6. Franchi MV, Monti E, Carter A, Quinlan JI, Herrod PJJ, Reeves ND, et al. Bouncing back! Counteracting muscle aging with plyometric muscle loading. Frontiers in physiology. 2019;10:178.

7. Ha A, Sum R, Chan C, Ng JYY. Promoting rope skipping at Hong Kong schools with low and mid socioeconomic statuses: An ecological perspective. International Review of Social Science. 2014 01/01;2:104-15.

8. Hunnicutt JL, Aaron SE, Embry AE, Cence B, Morgan P, Bowden MG, et al. The effects of POWER training in young and older adults after stroke. Stroke research and treatment. 2016;2016:7316250.

9. Keiner M, Sander A, Wirth K, Schmidtbleicher D. The impact of 2 years of additional athletic training on the jump performance of young athletes. Science and Sports. 2014;29(4):e39-e46.

10. Marina M, Jemni M. Plyometric training performance in elite-oriented prepubertal female gymnasts. J Strength Cond Res. 2014;28(4):1015-25.

11. Marta CC, Marinho DA, Izquierdo M, Marques MC. Differentiating maturational influence on training-induced strength and endurance adaptations in prepubescent children. American journal of human biology : the official journal of the Human Biology Council. 2014 Jul-Aug;26(4):469-75.

12. Michel M, Monem J, Ferran R. A two-season longitudinal follow-up study of jumps with added weights and counter movement jumps in well-trained pre-pubertal female gymnasts. J Sports Med Physical Fit. 2014;54(6):730-41.

13. Mero AMJ, Häkkinen K, Kyröläinen H, Mero AA. Effects of training on bone metabolism in young athletes. Human Movement. 2021;22(4):105-12.

14. Pardos-Mainer E, Ustero-Pérez O, Gonzalo-Skok O. Effects of upper and lower body plyometric training on physical performance in young tennis players. RICYDE: Revista Internacional de Ciencias del Deporte. 2017;13(49):225-43.

15. Pena-Gonzalez I, Fernandez-Fernandez J, Cervello E, Moya-Ramon M. Effect of biological maturation on strength-related adaptations in young soccer players. Plos One. 2019 Jul;14(7).

16. Radnor JM, Lloyd RS, Oliver JL. Individual response to different forms of resistance training in school-aged boys. J Strength Cond Res. 2017;31(3):787-97.

17. Saez de Villarreal E, Requena B, Arampatzi F, Salonikidis K. Effect of plyometric training on chair-rise, jumping and sprinting performance in three age groups of women. J Sports Med Phys Fitness. 2010 Jun;50(2):166-73.

18. Sáez Sáez de Villarreal E. Effect of plyometric training in three age groups of women. Rev Int Med Cienc Act Fis Dep. 2010;10(39):393-409.

19. Vassil K, Bazanovk B. The effect of plyometric training program on young volleyball players in their usual training period. J Human Sport Exerc. 2012;7:S35-S40.

20. Verma C, Subramanium L, Krishnan V. Effect of plyometric training on vertical jump height in high school basketball players: arandomised control trial. Int J Med Res Health Sci. 2015;4(1):7-12.
